# Does Malaria Cause Diarrhoea? A Systematic Review

**DOI:** 10.3389/fmed.2020.589379

**Published:** 2020-11-19

**Authors:** Isatou C. M. Sey, Ajoke M. Ehimiyein, Christian Bottomley, Eleanor M. Riley, Jason P. Mooney

**Affiliations:** ^1^Institute of Immunology and Infection Research, School of Biological Sciences, University of Edinburgh, Edinburgh, United Kingdom; ^2^Department of Veterinary Medicine, Ahmadu Bello University, Zaria, Nigeria; ^3^Department of Infectious Disease Epidemiology, London School of Hygiene and Tropical Medicine, London, United Kingdom

**Keywords:** malaria, plasmodium, diarrhoea, intestine, coinfection, systematic review

## Abstract

Malaria is a systemic febrile disease that may progress to prostration, respiratory distress, encephalopathy, anemia, and death. Malaria is also an established risk factor for invasive bacterial disease caused, in the majority of cases, by invasive enteropathogens and in particular by non-Typhoidal *Salmonella* (NTS). Whilst various malaria-related pathologies have been implicated in the risk of NTS bacteraemia in animal models, including intestinal dysbiosis and loss of gut homeostasis, clinical evidence is lacking. As a first step in gathering such evidence, we conducted a systematic review of clinical and epidemiological studies reporting the prevalence of diarrhoea among malaria cases and *vice versa*. Database searches for “plasmodium” and “diarrhoea” identified 1,771 articles; a search for “plasmodium” and “gastroenteritis” identified a further 215 articles. After review, 66 articles specified an association between the search terms and referred primarily, but not exclusively, to *Plasmodium falciparum* infections. Overall, between 1.6 and 44% of patients with acute malaria infection reported symptoms of diarrhoea (812 of 7,267 individuals, 11%) whereas 5–42% of patients presenting to hospital with diarrhoea had an underlying malaria parasite infection (totaling 749 of 2,937 individuals, 26%). However, given the broad range of estimates, a paucity of purposeful case control or longitudinal studies, and varied or poorly specified definitions of diarrhoea, the literature provides limited evidence to draw any firm conclusions. The relationship between malaria and gastrointestinal disturbance thus remains unclear. Carefully designed case-control studies and prospective longitudinal studies are required to confidently assess the prevalence and significance of intestinal manifestations of malaria parasite infection.

## Introduction

Malaria is a systemic, multi-organ disease characterized by fever, lethargy, headache, nausea, and muscle pains that may progress to prostration, respiratory distress, encephalopathy, anemia, and death (1). These symptoms result from the inflammatory response to the causative agent, *Plasmodium* spp., destruction of parasite-infected red blood cells (iRBC) and the ability of infected red blood cells to adhere to the endothelium of small blood vessels (2). Importantly, malaria is also an established risk factor for invasive bacterial disease caused, in the majority of cases, by invasive enteropathogens and in particular by non-Typhoidal *Salmonella* (NTS) (3, 4). Bacteraemia occurs in approximately 6.5% of severe malaria infections (4) and has a high case fatality rate (5).

Studies in laboratory animals have implicated a number of malaria-related pathologies in the increased risk of NTS bacteraemia including systemic neutrophil dysfunction (6), IL-10-mediated inhibition of antibacterial immunity (7), and intestinal dysbiosis and breakdown of gut homeostasis (8). Accordingly, mouse malaria infections have been associated with caecal inflammation (8), increased gut permeability (9), mononuclear infiltration (8, 9), and mastocytosis (10). *Plasmodium* spp. DNA can be detected in the feces of non-human primates (11, 12) suggesting that iRBC may be released during turnover of intestinal epithelium and an increased incidence of diarrhoea has been reported in Magellanic penguins (*Spheniscus magellanicus*) naturally infected with *Plasmodium* spp. (13). Together, these studies suggest that malaria infections may lead to intestinal pathology and that this may underlie the risk of systemic spread of enteropathogens.

There have been several reports of gastrointestinal symptoms, including nausea, vomiting, and diarrhoea, in patients with uncomplicated malaria (14–18) and diarrhoea is included in the World Health Organization list of malaria symptoms (19). However, as both uncomplicated malaria (20) and gastroenteritis (21) are common in malaria endemic regions of the world, as people in endemic areas may carry malaria parasites in their blood without any overt symptoms such that malaria can be an incidental finding in people with a variety of illnesses, and as several antimalarial drugs (including mefloquine, chloroquine, and doxycycline) can cause diarrhoea (1), it remains unclear whether malaria is a direct or indirect cause of diarrhoea or whether their occurrence is simply coincidental. For example, household clustering of reported malaria-like illness and all-cause diarrhoea in Malawi may reflect a causal relationship or simply shared and overlapping risk factors (22).

Although *Plasmodium* spp. infection alone can elicit mild inflammation (8) and dampen robust enteropathogen-induced intestinal responses (23) in rodents, there is little data on intestinal pathology in human malaria infections. Anecdotal reports from human autopsy studies include evidence of iRBC localization in intestinal mucosa with apparent sequestration of parasitized cells in villous capillaries (24, 25) and hemorrhages associated with the presence of iRBCs (26) but these represent only the most severe (i.e., fatal) malaria infections. The degree of intestinal pathology in mild malaria cases or in those with severe but non-fatal infections, and its relationship or not to the increased risk of bacteremia, thus remains undefined. As diarrhoea is a frequent consequence of intestinal inflammation, determining whether diarrhoea is a feature of *Plasmodium* spp. infection and/or malarial disease is a useful first step to understanding whether intestinal inflammation is either necessary or sufficient for bacterial translocation and subsequent bacteraemia.

To our knowledge, the evidence linking malaria infection to gastrointestinal symptoms was last reviewed in 1993 (27) and comprised only 7 small studies (numbers of subjects per study ranging from 1 to 26). We have therefore carried out a systematic review of the literature to estimate the prevalence of diarrhoea during acute clinical malaria infections with the aim of informing future research into the contribution of malaria-induced gastrointestinal disease in invasive bacterial disease and other comorbidities.

## Materials and Methods

### Search Strategy

Reports were retrieved through systematic searches of electronic databases using search terms “plasmodium” AND “diarrh^*^” (to accommodate various spellings of diarrhoea); for databases where ^*^ was not an allowed term, the search terms were “plasmodium” AND “diarrhoea” OR “Diarrhea.” Web of Science, Embase, Google Scholar, and PubMed were searched on the 23rd of January 2020 and the Global Health CABI database was searched on the 28th of January 2020, all in accordance with established guidelines (28). In addition, searches using the terms “plasmodium” AND “gastroenteritis” were performed on 20 August 2020 in PubMed, Web of Science, and Google Scholar. Articles in English, without any restriction on publication date, were imported into the Covidence systematic review software (Veritas Health Innovation, Melbourne, Australia). Given the very large number of hits with low relevance, and in line with best practice (28), only the first 200 articles (sorted by relevance) returned by Google Scholar were included.

### Article Screening

Titles and abstracts were independently screened for inclusion or exclusion by two reviewers (IS and JM), with conflicts requiring discussion and unanimous resolution. Key words for initial inclusion included; “malaria”, “plasmodium,” “diarrhoea,” “Diarrhea,” “dysentery,” “gut,” “intestine”; keywords for exclusion included “drug trial” and “adverse event,” as medication-induced diarrhoea was not of interest here. Full-texts of all provisionally included studies were obtained via The University of Edinburgh library DiscoverEd service, including both electronic and print editions. Articles were eligible for final inclusion if they met the following criteria: (1) had confirmed the diagnosed infection with *Plasmodium* spp. using microscopy, a rapid diagnostic test (RDT) or PCR (polymerase chain reaction), (2) reported on the prevalence or incidence of diarrhoea during an episode of clinical malaria, (3) were confirmed to be in English, and (4) the full text article was available for review. As a further check for consistency, at the end of the process 50 articles were randomly selected from the excluded pool of papers and re-reviewed (Supplementary Table 1).

### Data Extraction and Meta-Analysis

The following variables were extracted: country, study site, sample size, diagnostic methods used for malaria (i.e., microscopy, RDT, or PCR), *Plasmodium* species, proportion with malaria and diarrhoea, age and sex of participants, comorbidities, and equivalent data for controls (where applicable). Manuscripts were classified as Group 1: case reports; Group 2: diarrhoea reported secondary to malaria infection; and Group 3: reports of diarrhoea in which malaria prevalence was recorded. Case reports in Group 1 were qualitatively assessed. The overall prevalence of diarrhoea in individuals with malaria was estimated by combining subject level data from studies in Group 2 and the prevalence of malaria in individuals with diarrhoea was estimated by combining subject level data from studies in Group 3. Where data were available from a suitable control group without the presenting condition, odds ratios were calculated. Extracted data were organized and graphed using Microsoft excel and GraphPad Prism 8.

## Results

**Figure 1:**
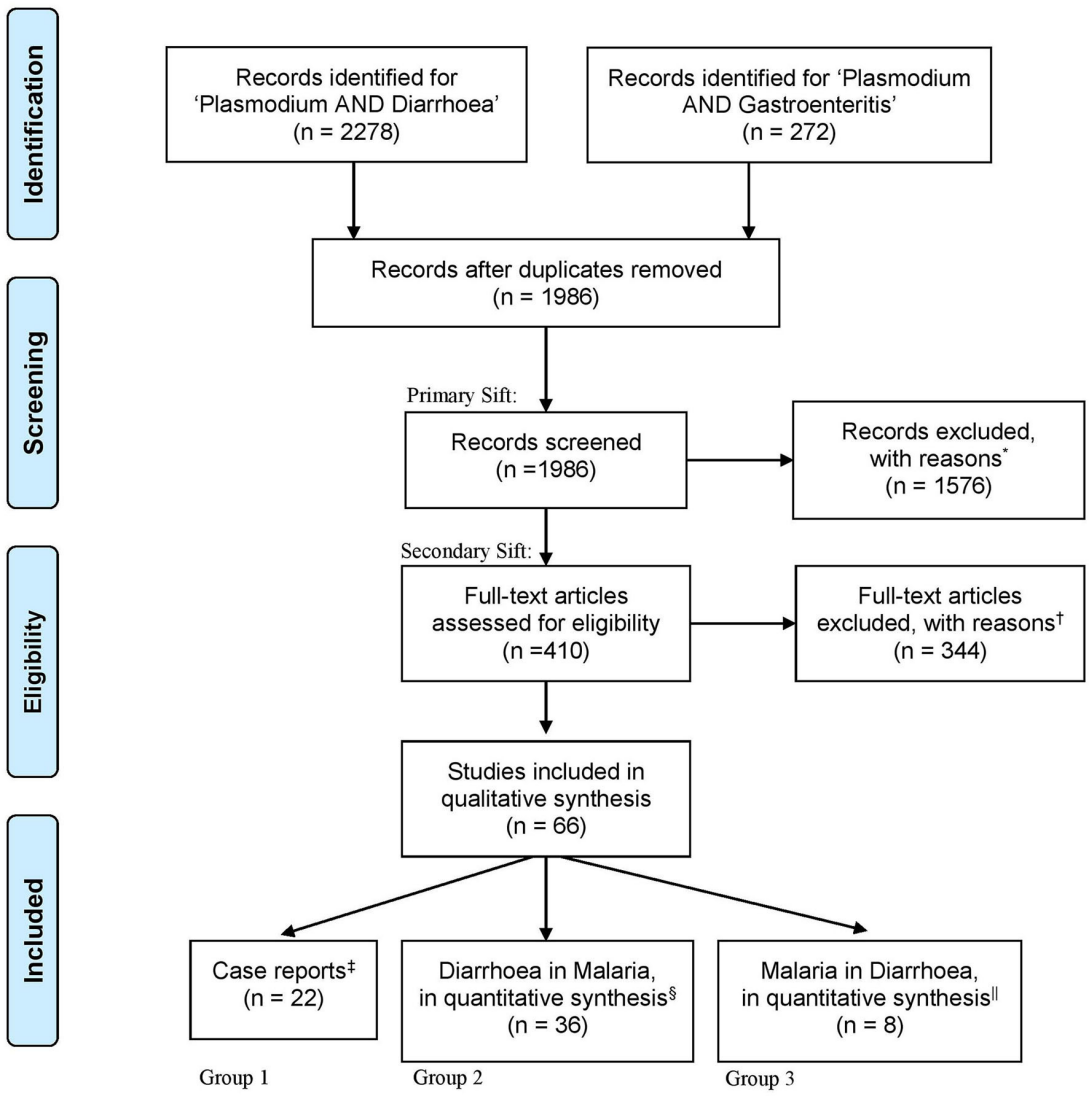
An illustration of the search process, as outlined by the “Preferred Reporting Items for Systematic Reviews and Meta-Analyses (PRISMA).” Following database searches for “Plasmodium AND Diarr*,” “Plasmodium AND Diarrhoea OR Diarrhea,” or “Plasmodium AND Gastroenteritis” a total of 2,550 articles were identified. One relevant article was added which was not found by the search scheme (29). All abstracts were imported into Covidence software for subsequent double-blind review. Five hundred sixty-four duplicates were removed, leaving 1,986 for title and abstract screening. Articles were included if keywords were mentioned in the title or abstract; including “malaria,” “Plasmodium,” “diarrhoea,” “dysentery,” “gut,” and “intestine.” Articles were then grouped according to study type and primary presenting syndrome. *Of the 1,576 excluded articles, 50 were randomly re-reviewed for exclusion, with reasons reported in Supplementary Table 1. Four hundred ten full-text articles were screened and 344 were excluded, with reasons reported in Supplementary Table 2. In all, 66 articles qualified for data extraction. ^‡^Case reports accounted for 22 articles. ^§^Thirty-six articles reported diarrhoea secondary to the presentation of malaria parasite infection. ^||^Eight articles reported malaria as a condition underlying the presentation of diarrhoea.

In total, 1,986 unique records were identified using the search terms “plasmodium” and “diarrhoea” or “plasmodium” and “gastroenteritis” (Figure 1). Of these, 410 articles met the criteria for provisional inclusion for full text review (primary sift) and, of these, 22 case reports (Group 1) and 44 research articles (36 in Group 2 and 8 in Group 3) met the criteria for final inclusion (secondary sift) (Figure 1). Examples of articles excluded at the primary sift are shown in Supplementary Table 1 and a summary of the reasons for exclusion of articles at the secondary sift is given in Supplementary Table 2.

**Figure 2:**
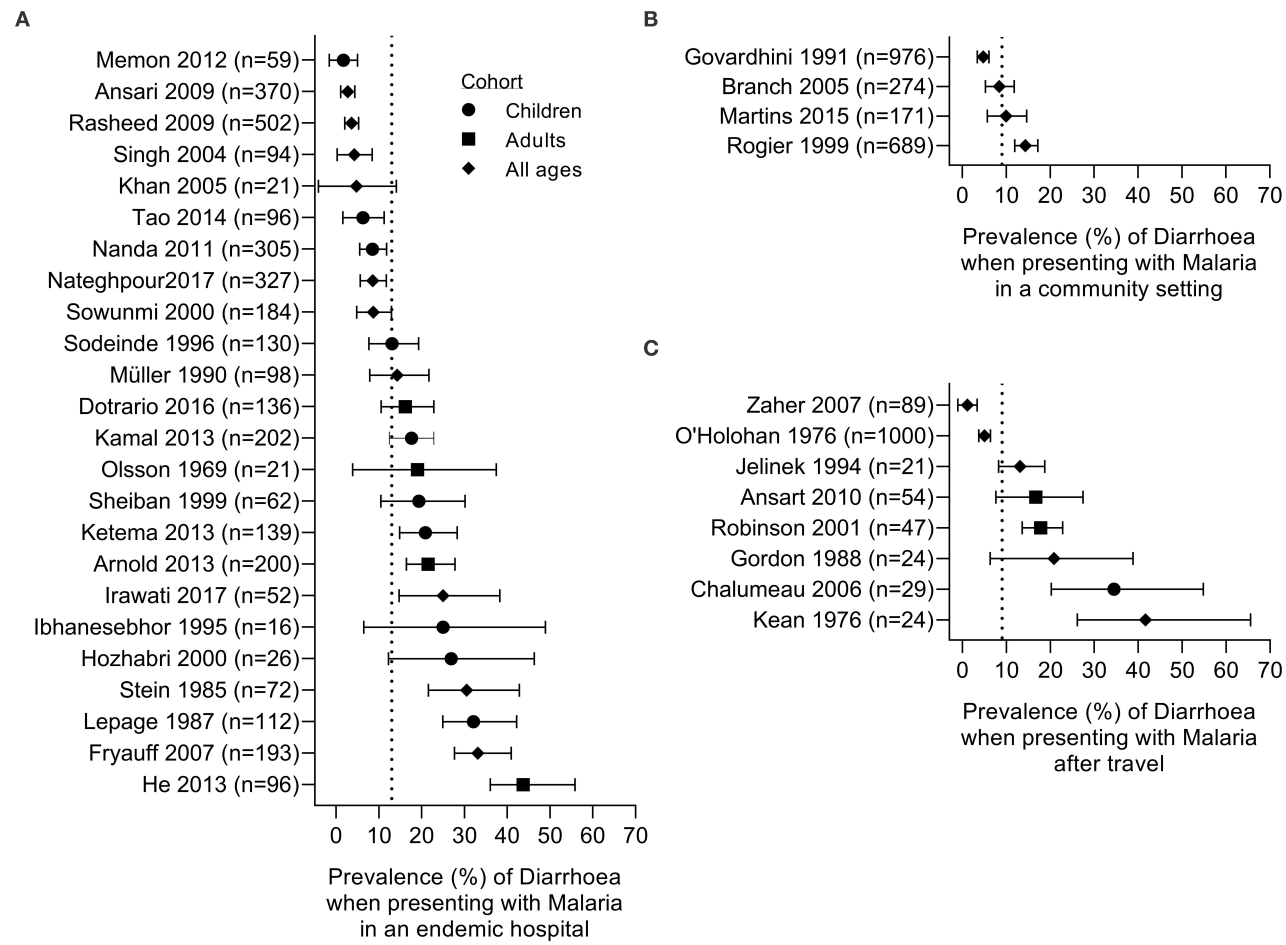
Prevalence of diarrhoea in subjects with malaria. Studies are grouped by setting: **(A)** patients seeking care in a hospital setting in an endemic area, **(B)** community survey; **(C)** malaria in travelers admitted to hospital on their return home. Dotted line represents the prevalence estimate from the combined data: **(A)** 13%, **(B)** 9%, and **(C)** 9%. Symbols denote the age of the studied cohort: children (circle), adults (square), or all ages (diamond).

**Figure 3:**
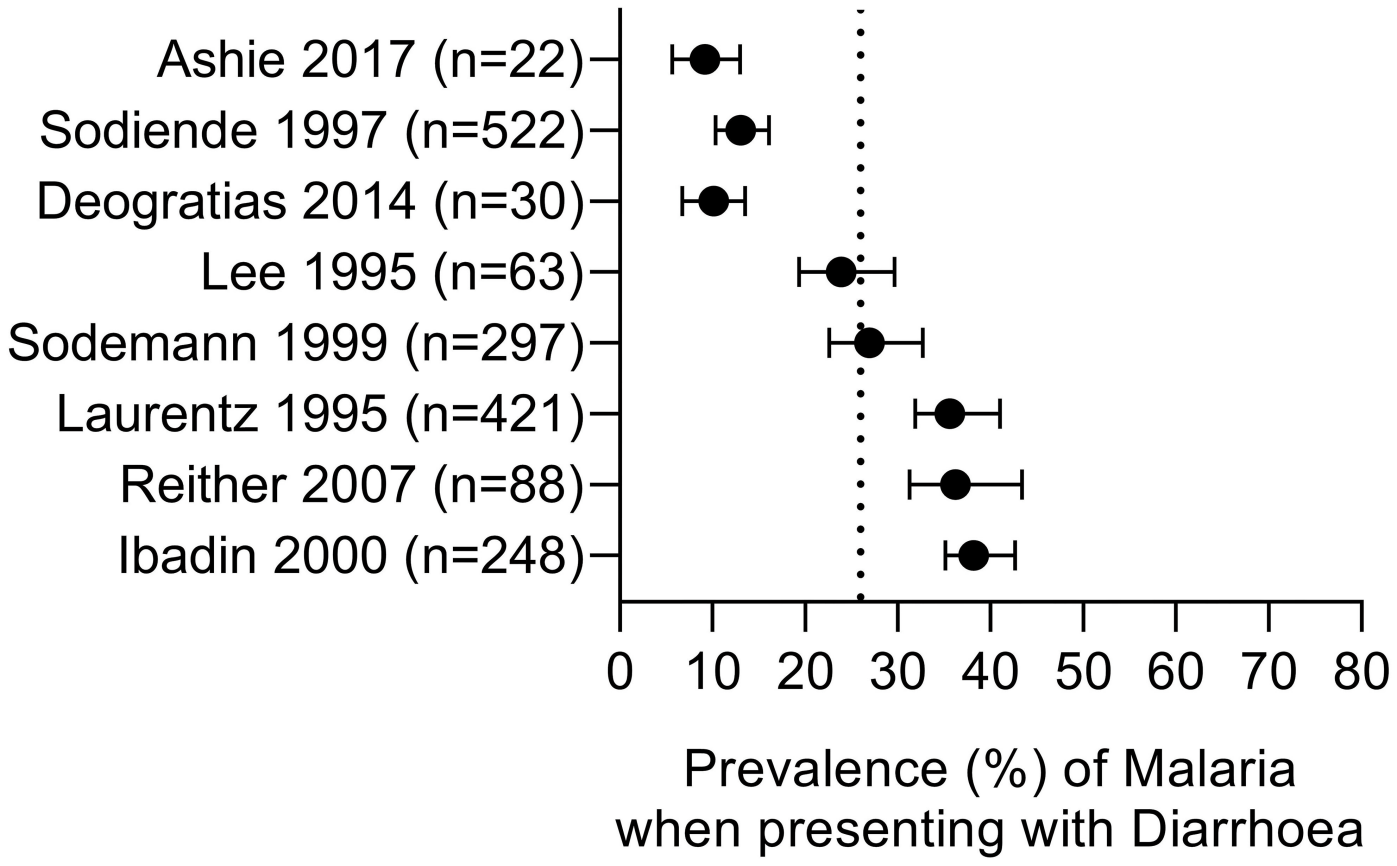
Prevalence of malaria parasitaemia in patients with diarrhoea. Summary of studies where diarrhoea was the primary presentation and malaria parasite infection was subsequently ascertained. Dotted line represents the prevalence estimate from the combined data (26%). All studies were conducted in children.

Qualitative data were extracted from case reports for residents of malaria endemic areas (*n* = 7 reports; Supplementary Table 3) and for travelers (*n* = 15 reports; Supplementary Table 4). Quantitative analysis of diarrhoea prevalence was performed using data from cohort studies in endemic areas where diarrhoea was reported at the time of presentation to a health care facility with a primary diagnosis of malaria (*n* = 24 studies; Table 1 and Figure 2A), among travelers to malaria endemic areas presenting at hospital with a primary diagnosis of malaria (*n* = 8 studies; Table 2 and Figure 2B) and on data captured during community surveys (*n* = 4 studies; Table 3 and Figure 2C). Finally, the prevalence of *Plasmodium* infection was analyzed among individuals where diarrhoea was the primary clinical presentation at a health care facility (*n* = 8 studies; Table 4 and Figure 3). Where prevalence estimates were reported in controls, odds ratios (OR) were calculated (Tables 5, 6).

**Table 1:** Prevalence of diarrhoea in patients who present in hospital with malaria in a malaria-endemic area.

**References**	**Origin**	**Patient(s)**	**Study Location**	**Case Control Study?**	**Patient recruitment**	**Retrospective/ Prospective**	**Malaria parasite diagnosis**		**Definition of diarrhoea**	**Patients with malaria, *n***	**Patients reporting diarrhoea *n*, (%)**	**Comments**
							**Method**	**Species**				
Olsson and Johnston (30)	Vietnam	Adults, males (soldiers)	Hospital	No	Passive	Prospective	Microscopy	*P. falciparum*	2–4 watery stools/day	21	4 (19%)	Data includes exploratory biopsies with pathology
Stein and Gelfand (31)	Zimbabwe	Children (52%) and adults, males (51%), and females	Hospital	No	Passive	Retrospective	Microscopy	*P. falciparum*	None	72	22 (30.6%)	–
Lepage et al. (32)	Rwanda	Children	Hospital	Yes	Passive	Prospective	Microscopy	*P. falciparum*	None	112	36 (32.1%)	Data from febrile, non-bacteraemic P.f. children as “controls,” Group II
Müller and Moser (33)	Uganda	Children and adults	Hospital	Yes	Passive	Retrospective	Microscopy	*P. falciparum*	None	98	14 (14.2%)	Data from HIV neg “controls” with diarrhoea >1 month (Tables 3, 6)
Ibhanesebhor (34)	Nigeria	Neonates (mean 38 weeks)	Hospital	No	Passive	Prospective	Microscopy	*P. falciparum*	None	16	4 (25%)	Table 1
Sodeinde et al. (35)	Nigeria	Children	Hospital	No	Passive	Prospective	Microscopy	*P. falciparum*	3 or more loose bowel motions in the preceding 24 h	130	17 (13.1%)	Data from cerebral malaria patients at two sites, Table 1
Sheiban (36)	Yemen	Children	Hospital	No	Passive	Prospective	Microscopy	*P. falciparum*	None	62	12 (19.4%)	Data from deaths and survivors with renal failure
Sowunmi et al. (37)	Nigeria	Children and teenagers	Hospital	No	Passive	Prospective	Microscopy	*P. falciparum*	4 or more loose or watery stools /day for at least 1 day	184	16 (8.7%)	Data from acute symptomatic infection, mean duration of diarrhoea 1.8 days
Hozhabri et al. (38)	Pakistan	Children (median 24 mo)	Clinic	Yes	Passive	Prospective	Microscopy	*P. falciparum*	None	26	7 (26.9%)	Table 2
Singh et al. (39)	Malaysia	Children and adults (mean 35 y)	Hospital	No	Passive	Prospective	Microscopy and PCR	*P. knowlesi*	None	94	4 (4.3%)	Data from Kapit Hospital only, Table 2
Khan et al. (40)	Pakistan	Children and adults (median 5 y)	Hospital	Yes	Passive	Retrospective	Microscopy	*P. falciparum, P. vivax*	None	21	1 (4.7%)	Data from “control” malaria without Typhoid fever, Table 2
Fryauff et al. (41)	Ghana	Children (mean 15 mo)	Community	No	Active	Prospective	Microscopy	*P. falciparum*	None	193	64 (33.2%)	Intervention and longitudinal study
Ansari et al. (42)	Pakistan	Teenagers and adults (mean 34 y)	Hospital	No	Passive	Prospective	Microscopy	*P. falciparum*	None	370	10 (2.7%)	–
Rasheed et al. (43)	Pakistan	Children and adults (mean 28 y)	Hospital	No	Passive	Prospective	Microscopy	*P. falciparum, P. vivax*, mixed infections	None	502	18 (3.6%)	Data from all *Plasmodium* spp., Table 1
Nanda et al. (44)	India	Children	Hospital	No	Passive	Retrospective	Microscopy	*P. falciparum*	None	305	26 (8.5%)	Duration of illness, 1–6 days
Memon et al. (45)	Pakistan	Children	Hospital	No	Passive	Prospective	Microscopy	*P. falciparum, P. vivax*	None	59	1 (1.6%)	–
Ketema and Bacha (46)	Ethiopia	Children (median 4 y)	Health Center	No	Passive	Retrospective	Microscopy	*P. vivax*	None	139	29 (20.9%)	Table 1
Kamal (47)	Pakistan	Children (median 7 y)	Hospital	No	Passive	Prospective	Microscopy	*P. falciparum, P. vivax*	None	202	35 (17.3%)	–
Arnold et al. (48)	Thailand	Adults	Hospital	No	Passive	Retrospective	Microscopy	*P. falciparum*	None	200	43 (21.5%)	Data from severe malaria patients, with or without shock
He et al. (49)	South Sudan	Adults, soldiers	Hospital	No	Passive	Prospective	Microscopy and/or RDT	*P. falciparum*	None	96	42 (43.8%)	Data from no/incomplete chemoprophylaxis group, this table
Tao et al. (50)	China	Neonates	Unclear	No	Passive	Retrospective	Microscopy	*P. vivax, P. falciparum, P. malariae*	None	96	6 (6.3%)	Data from clinical case reports in database, presumably hospital records
Dotrário et al. (51)	Brazil	Adults (median 37 y)	Hospital	Yes	Passive	Retrospective	Microscopy	*P. vivax, P. falciparum, P. malariae*, mixed infections	None	136	22 (16.2%)	Table 3, intervention and longitudinal study
Irawati et al. (52)	Indonesia	Children and adults	Community Survey	No	Active	Prospective	Microscopy	*P. falciparum*	None	52	13 (25%)	Table 2, intervention and longitudinal study
Nateghpour et al. (53)	Iran	Children and adults	Health Center	No	Passive	Prospective	Microscopy	*P. falciparum, P. vivax*, mixed infections	None	327	28 (8.6%)	Table 2
Sum of data taken from all studies in table										3,513	474 (13%)	

**Table 2:** Prevalence of diarrhoea in patients who present with malaria after traveling to a malaria-endemic area.

**References**	**Origin of infection**	**Country of diagnosis**	**Patient(s)**	**Study location**	**Case control study?**	**Patient recruitment**	**Retrospective/ Prospective**	**Malaria parasite diagnosis**		**Definition of diarrhoea**	**Patients with malaria, *n***	**Patients reporting diarrhoea *n*, (%)**	**Comments**
								**Method**	**Species**				
O'Holohan (54)	Malaysia	Malaysia	Children and adults	Clinic	No	Passive	Retrospective	Microscopy	*P. falciparum, P. vivax*, mixed infections	None	1,000	50 (5%)	Table 5
Kean and Reilly (55)	West Africa, East Africa, SE Asia, and the Caribbean	USA	Children and adults (mean 33 y)	Hospital	No	Passive	Retrospective	Microscopy	*P. falciparum P. vivax, P. malariae, P. ovale*, mixed infections	None	24	10 (41.67%)	Table 4
Gordon et al. (56)	India, Pakistan, El Salvador, Guatemala, Nigeria, Sierra Leone, Haiti	USA	Children and adults (mean 28 y)	Hospital	No	Passive	Retrospective	Microscopy	*P. vivax, P. falciparum*	None	24	5 (20.8%)	Table 1
Jelinek et al. (57)	Africa, Asia, Central South America	Germany	Children and adults (median 32 y)	Travel clinic	No	Passive	Unclear	Microscopy	*P. falciparum, P. vivax, P. ovale, P. malariae*, mixed infections	None	160	21 (13.13%)	–
Robinson et al. (58)	Africa, Asia, the Americas, Solomon Islands	Australia	Adults (median 29 y)	Hospital	No	Passive	Retrospective	Microscopy	*P. vivax, P. falciparum, P. malariae, P. ovale*, mixed infections	None	264	47 (17.8%)	Table 2
Chalumeau et al. (59)	Africa	France	Children (median 10 y)	Hospital	No	Passive	Prospective	Microscopy	*P. falciparum*	None	29	10 (34.48%)	Table 1
Zaher et al. (60)	Pilgrimage (various)	Yemen, Egypt, Saudi Arabia	Teenagers and adults	Hospital	No	Passive	Prospective	Microscopy	–	None	89	1 (1.12%)	Table 3
Ansart et al. (61)	Africa, Asia, USA, the Caribbean	France	Adults	Hospital	No	Passive	Prospective	Microscopy	*P. falciparum, P. vivax, P. ovale*	None	54	9 (16.67%)	Table 4
Sum of data taken from all studies in table											1,644	153 (9%)	

**Table 3:** Prevalence of diarrhoea in patients who present with malaria in a malaria-endemic community setting.

**References**	**Origin**	**Patient(s)**	**Study location**	**Case control study?**	**Patient recruitment**	**Retrospective/ Prospective**	**Malaria parasite diagnosis**		**Definition of diarrhoea**	**Patients with malaria, *n***	**Patients reporting diarrhoea *n*, (%)**	**Comments**
							**Method**	**Species**				
Govardhini et al. (62)	India	Unclear age, sex	Community	Yes	Passive	Prospective	Microscopy	*P. falciparum*	None	976	46 (4.71%)	Table 1
Rogier et al. (79)	Senegal	Children and teenagers	Community	No	Active	Prospective	Microscopy	*P. falciparum*	Increased number (usually 3/day) of liquid stool	689	99 (14.37%)	Table 1
Branch et al. (80)	Peru	Children and adults	Community	No	Active	Prospective	Microscopy	*P. falciparum, P. vivax*	None	274	23 (8.39%)	–
Martins et al. (81)	Brazil	Children and adults (median 26y)	Community	No	Passive	Prospective	Microscopy	*P. falciparum, P. vivax*	None	171	17 (9.94%)	Table 2
Sum of data taken from all studies in table										2,110	185 (9%)	

**Table 4:** Prevalence of malaria in patients with underlying diarrhoea.

**References**	**Origin**	**Patient(s)**	**Study location**	**Case control study?**	**Patient recruitment**	**Retrospective/ Prospective**	**Malaria parasite diagnosis**		**Definition of diarrhoea**	**Patients with diarrhoea, *n***	***N* (%) of diarrhoea patients with malaria parasitaemia**	**Comments**
							**Method**	**Species**				
Laurentz and Manoempil (63)	Indonesia	Children	Hospital	No	Passive	Prospective	Microscopy	*P. falciparum*	A condition of frequent watery stool	421	150 (35.6%)	Table 1
Lee et al. (66)	Ivory Coast	Children	Hospital	No	Passive	Prospective	Microscopy	*P. falciparum*	Diarrhoea for >3 days	264	63 (23.9%)	Table 2, both severe and non-severe illness
Sodeinde et al. (29)	Nigeria	Children (median 15 mo)	Hospital	Yes	Passive	Prospective	Microscopy	*P. falciparum*	None	522	68 (13%)	Table 2
Sodemann et al. (64)	Guinea-Bissau	Children (median 19 mo)	Community	Yes	Active	Prospective	Microscopy	*P. falciparum*	3 or more stools in 24 h, and only for <3 days	297	80 (26.9%)	Table 1
Ibadin et al. (67)	Nigeria	Children (mean 14 mo)	Hospital	No	Passive	Retrospective	Microscopy	*P. falciparum*	3 or more watery or liquid stools within 24 h	650	248 (38.2%)	Table 1
Reither et al. (68)	Ghana	Children (mean 0.8 y)	Hospital	Yes	Active	Prospective	Microscopy and PCR	*P. falciparum*	3 or more watery or loose stools within 24 h for <14 days	243	88 (36.2%)	Table 1
Deogratias et al. (69)	Tanzania	Children (median 1 y)	Hospital	No	Passive	Prospective	Microscopy	*P. falciparum*	3 or more abnormally loose or fluid stools in the past 24 h; with or without dehydration	300	30 (10%)	Table 1
Ashie et al. (65)	Ghana	Children (<5 y)	Hospital	Yes	Passive	Prospective	Microscopy	Not stated but likely to be predominantly *P. falciparum*	3 or more watery or loose stools within 24-h period prior to admission to the hospital	240	22 (9.2%)	Table 2
Sum of data taken from all studies in table										2,937	749 (26%)	

**Table 5:** Odds ratio of presenting with diarrhoea secondary to malaria, when compared to “controls” without malaria.

**References**	**Origin**	**Patient(s)**	**Malaria-infected**			**“Controls”—Malaria negative**				**Odds Ratio (95% CI)**
			**Species**	**Number with Malaria, *n***	**Diarrhoea positive, *n* (%)**	**Control recruitment**	**Fever?**	**Number of Malaria negative controls, *n***	**Controls with diarrhoea, *n* (%)**	
Lepage et al. (32)	Rwanda	Children	*P. falciparum*	112	36 (32%)	Hospital	Yes, febrile	112	26 (23%)	1.57 (0.87, 2.82), *p* = 0.14
Govardhini et al. (62)	India	Unclear age, sex	*P. falciparum*	976	46 (4.7%)	Community	Both febrile and afebrile	4,143	160 (3.9%)	1.23 (0.88, 1.72), *p* = 0.22
Hozhabri et al. (38)	Pakistan	Children (median 24 mo)	*P. falciparum*	26	7 (26.9%)	Hospital	Yes, febrile	412	99 (24.0%)	1.16 (0.49, 2.79), *p* = 0.74
Dotrário et al. (51)	Brazil	Adults (median 37 y)	*P. vivax, P. falciparum, P. malariae*, mixed infections	136	22 (16.2%)	Hospital	Yes, febrile	157	52 (33.1%)	0.39 (0.22, 0.68), *p* = 0.001

**Table 6:** Odds ratio of having underlying malaria when presenting with diarrhoea, compared to “controls.”

**References**	**Origin**	**Patient(s)**	**Cases—with diarrhoea**			**“Controls”—without diarrhoea**				**Odds ratio (95% CI)**
			**Number with diarrhoea, *n***	**Malaria positive, *n* (%)**	**Species**	**Control recruitment**	**Fever?**	**Number of diarrhoea negative controls, *n***	**Controls with malaria, *n* (%)**	
Laurentz and Manoempil (63)	Indonesia	Children	821	150 (18.3%)	*P. falciparum*	Hospital	69% febrile	1,192	271 (22.7%)	0.76 (0.61, 0.95), *p* = 0.02
Sodeinde et al. (29)	Nigeria	Children (median 15 mo)	522	68 (13%)	*P. falciparum*	Hospital	75% febrile	313	56 (17.9%)	0.69 (0.46, 1.01), *p* = 0.056
Sodemann et al. (64)	Guinea-Bissau	Children (median 19 mo)	297	80 (26.9%)	*P. falciparum*	Community	15% febrile, via maternal reporting	297	72 (24.2%)	1.15 (0.80, 1.66), *p* = 0.45
Ashie et al. (65)	Ghana	Children (<5 y)	240	22 (9.2%)	Not stated but likely to be predominantly *P. falciparum*	Hospital	Not reported	107	1 (0.9%)	10.7 (1.42, 80.4), *p* = 0.02

### Qualitative Findings—Case Reports

A third of articles found (22/66; 33%) were descriptive reports of diarrhoea in patients presenting with malaria, either living in or with recent travel to malaria endemic areas (Supplementary Tables 3, 4). Seven papers describe a total of 8 cases (aged 8–69 years) from endemic populations. Six papers reported a history of diarrhoea prior to arrival at hospital (with a duration of 2 days to 4 weeks) and patients were diagnosed with either *P. falciparum* (*n* = 4), *P. malariae* (*n* = 2), or *P. vivax* (*n* = 1) malaria. One case of constipation lasting 4 days, and resolving after antimalarial treatment, was reported in a Cameroonian patient with *P. falciparum* malaria (70). Coinfections were frequent in this group, with schistosomiasis and leishmaniasis (71), borreliosis (72), and *Salmonella* Typhi (73) infections complicating the attribution of diarrhoea. Of particular concern, a fatal outcome was reported for a patient from Sarawak who presented with epigastric pain, fever, vomiting and diarrhoea and was diagnosed with acute gastroenteritis; only after failure to respond to treatment was a malaria blood film examined, revealing >750,000 *P. malariae* iRBC per microliter of blood (74).

Fifteen papers reported cases among 20 travelers (aged 3–84 years) from malaria non-endemic to endemic areas, of whom 16 reported diarrhoea in the presence of malaria (8 *P. falciparum*, 4 *P. vivax*, 1 *P. knowlesi*, and 3 *P. ovale*). In most cases, diarrhoea was associated with onset of other symptoms of malaria, with duration of between 1 and 14 days prior to seeking treatment (median 3 days). In one case, diarrhoea was persistent (4 months) but likely associated with underlying HIV infection and coccidiosis rather than the acute malaria episode (75) and in another case, the onset of bloody diarrhoea 2 days after admission to hospital with severe malaria was likely associated with coinfection with Crimean Haemorrhagic Fever (76). Gross pathology by endoscopy of the duodenum showed a thick irregular mucosa with multiple erosions in a patient with *P. falciparum* and acute onset bloody diarrhoea (77).

Taken together, these case reports suggest that acute onset abdominal pain and/or diarrhoea may accompany the onset of other malarial symptoms and may be a feature of infection with any of the human-infective *Plasmodium* spp.; in severe cases, this may obscure or delay the diagnosis of malaria, with potentially fatal outcome. Although, by their very nature, case reports tend to report unexpected or atypical findings and cannot be relied upon for drawing generalizable conclusions (78), these case reports do reveal that diarrhoea associated with malaria—especially when severe and/or bloody—may be indicative of a concomitant infection that warrants further clinical investigations.

### Quantitative Findings—Diarrhoea Associated With Acute Malaria Episodes

We identified 32 health facility-based studies (24 in endemic settings and 8 in non-endemic settings) and 4 community studies of acute malaria cases in which the prevalence of diarrhoea was reported. In all but two studies, malaria diagnosis, and speciation were performed solely by microscopy and definitions of diarrhoea were provided for only 4 studies. The 32 studies comprised 7,267 individuals, of whom 812 (11%) were reported to have diarrhoea (Tables 1–3). Variation in prevalence of diarrhoea by age, sex, *Plasmodium* species, or severity of disease cannot be confidently assessed with the data available, as discussed in detail below.

Of the 24 hospital-based studies in endemic areas, five were case-control studies with the remainder being cohort studies of varying design (Table 1). Nine studies were from Africa, 7 from the Indian subcontinent, 5 from east and southeast Asia, 2 from the Middle East and 1 from Latin America. In 15 studies, *P. falciparum* was the only malaria parasite reported; 5 studies reported both *P. falciparum* and *P. vivax* cases with some mixed infections, 2 studies reported *P. falciparum, P. vivax* and *P. malariae* infections, whereas 1 study each reported *P. vivax* or *P. knowlesi* cases only. The prevalence of diarrhoea ranged from 1.6% [in a study of 59 children from Pakistan; (45)] to 43.8% [among 96 adults from South Sudan; (49)], with an overall prevalence of 13.5% (474 reports of diarrhoea among 3,513 malaria cases) (Table 1 and Figure 2A). Two case-control studies reported prevalence of diarrhoea in febrile patients with or without a *Plasmodium* positive blood smears in the context of either HIV (33) or Typhoid (40) infections. Four studies included febrile patients who were either malaria positive or malaria negative by microscopy (32, 38, 51, 62); in three of these studies the odds ratio of presenting with diarrhoea secondary to malaria was not significantly different from 1 whereas in one study the OR of 0.39 (95% CI 0.22, 0.68) was suggestive of a lower prevalence of diarrhoea in malaria cases compared to malaria uninfected patients (Table 5). Taken together, these data suggest that diarrhoea is not more common among malaria patients than among patients attending hospital for other reasons. However, this does rule out that diarrhoea may be more common among people with malaria than among malaria-uninfected people at a community level.

The 8 studies of travelers to malaria endemic areas who subsequently reported to hospitals in non-malaria-endemic regions comprised a total of 1,644 people, of whom 153 (9%) reported diarrhoea (Table 2 and Figure 2B). Prevalence within studies ranged from 1.1% [in a study of 89 returning pilgrims to Yemen and Egypt (60)] to 41.7% [in a study of 24 patients presenting at a New York City hospital (55)]. As might be expected, these case series tended to include people of all ages, traveling from different countries and infected with any of the human-infective malaria species. There will, of course, be many differences, including differences in immunity to malaria and gastrointestinal pathogens, between travelers and endemic populations that may affect their likelihood of developing diarrhoea during a malaria infection and data from travelers cannot be taken to infer what might be seen in endemic communities. Nevertheless, comparing data from travelers and residents of endemic areas may help to identify risk factors for diarrhoea associated with malaria infection.

Somewhat surprisingly, we identified only 4 studies that reported the prevalence of diarrhoea in community surveys of malaria (Table 3 and Figure 2C) and in only 1 of these studies was the prevalence of diarrhoea reported in malaria-negative cases. These were all prospective studies in children and teenagers and, in two cases, also in adults and reported *P. falciparum* and *P. vivax* cases. Prevalence of diarrhoea ranged from 4.7% [in a study of 976 children and teenagers in India (60, 62)] to 14.4% [in a study of 689 villagers of all ages in Senegal (79)], with an overall prevalence of 9% (185 reports of diarrhoea among 2,110 malaria cases).

### Quantitative Findings—Prevalence of Malaria in Patients Presenting With Diarrhoeal Disease

We identified 8 studies where malaria prevalence was recorded for patients presenting with a primary diagnosis of diarrhoea; all of the studies are in children, 7 studies are from Africa with one from Indonesia and all were related to *P. falciparum* infections (Table 4 and Figure 3). Three of the studies were cross-sectional studies of diarrhoea patients, 3 were prospective studies to investigate the prevalence of malaria during episodes of diarrhoea and 2 were case-control studies of patients with or without diarrhoea. The reported prevalence of malaria parasitaemia among patients with diarrhoea ranged from 9.2% [in a study of 240 children from Ghana (65)] to 38.2% [in a study of 650 children from Nigeria (67)], with an overall prevalence across all 8 studies of 26% (749 of 2,937 diarrhoeal cases) (Table 4). Four studies reported malaria prevalence in a control group to determine whether patients with diarrhoea were more or less likely to have malaria parasitaemia (Table 6). Sodemann et al. drew controls from the community and found no difference in malaria prevalence in individuals with or without diarrhoea (64). The other three studies were drew their controls from hospital admissions, consisting largely of febrile patients [69% (63) and 75% (29)]. In the study by Ashie et al., Ghanaian children under the age of 5 years presenting with a history of 3 or more watery or loose stools within the preceding 24 h, were 12-fold more likely to have concomitant malaria parasitaemia (adjusted OR = 10.7, 95% CI 1.42, 80.4, *p* = 0.02) than age-matched subjects attending the clinic for other reasons and with no history of diarrhoea for at least 21 days (65). However, the extremely low prevalence of malaria in the hospitalized control group (0.9%) in this study raises concerns, suggesting some form of bias as a contemporaneous studies from the same location (Kumasi) showed a high prevalence of malaria in this age group [52% in 2017 (82)] with relatively stable malaria prevalence since 2010 (83). Recognizing the potential impact of the easy availability of antimalarial drugs in many malaria-endemic communities on such studies, Sodemann et al. cautioned that their failure to find any association between diarrhoea and parasite rate, parasite density or clinical malaria in a nested case-control study in Guinea-Bissau, where the overall prevalence of malaria parasites was a surprisingly low 0.7%, did not preclude that diarrhoea may be a sign of clinical malaria (64). Lastly, both Sodeinde et al. (35) and Ibadin et al. (67) found that malaria parasitemia was more prevalent in diarrhoeal patients who were dehydrated; diarrhoea was reported as acute and watery rather than persistent and bloody (67). The extent of dehydration may be an indirect measure of the severity of diarrhoea and needs to be recorded since the consequent haemoconcentration may distort the calculation of parasitaemia unless adjusted for erythrocyte count.

## Discussion

This review has revealed widely varying estimates of the relationship between malaria infection and diarrhoea. Whilst this may reflect genuine differences in the impact of malaria infection on intestinal health, it may also reflect limitations in the way in which the search was carried out (our search was driven by the presence of search terms in the title, keywords or abstract favoring identification of studies where an association was found and was a significant conclusion of the study) and in the way data were collected (with very few appropriately designed studies on which to base robust estimates of any association). Given that the presentation of malaria is so non-specific, it is surprising how few studies (44 of 1,986 eligible studies; 2.2%) actually reported the prevalence of diarrhoea, raising the distinct possibility of publication bias. Furthermore, a definition of diarrhoea was provided in only 10 (23%) of these 44 studies, meaning that there may also have been considerable ascertainment bias. Self-reported diarrhoea is highly subjective, reflecting a deviation from what is considered normal for that person (84) or in that community (85, 86) and may be associated with differences in gut microbiota species richness (87). Finally, very few of the studies compared the prevalence of diarrhoea in malaria patients with that in controls or vice versa. Given that both malaria and diarrhoea are very common in many malaria endemic communities (35), may show similar seasonal variation associated with seasonal rains (62), and that people in endemic areas may carry malaria parasites in their blood without any overt symptoms such that malaria can be an incidental finding in a variety of illnesses, it is important to rule out coincidental occurrence of these two syndromes. Only four of the studies included in this review attempted to rule out other infectious causes of diarrhoea with stool cultures [(66, 68, 69); Table 4], and only one of these included stool cultural analysis from appropriate controls (65). Although anecdotal, given it is a single study, Reither et al. (68) found that *P. falciparum* infection occurred at similar prevalence in diarrhoeic patients with and without a clearly identified cause of diarrhoea but that malaria was more frequently symptomatic in children without an identified enteropathogen.

Although much of the data on the relationship between diarrhoea and malaria is difficult to interpret, it is notable that none of the 4 studies comparing the prevalence of diarrhoea in malaria cases and controls found evidence of a significant positive association (Table 5), suggesting that diarrhoea may not be causally associated with febrile malaria. However, these were all studies where the selection of controls is in itself highly problematic given that diarrhoea can be a genuine symptom of many illnesses leading to presentation at hospital. Longitudinal, community-based studies with clear definitions of both clinical malaria and diarrhoea are needed to properly ascertain the relationship between these two syndromes. It is likely that many prospective studies of malaria-associated illness, including large, carefully controlled intervention studies, have collected data on symptoms including diarrhoea but have not reported the data in such a way that any association can be inferred. For example, a trial of seasonal malaria chemoprophylaxis conducted in The Gambia included a longitudinal control group who did not receive prophylaxis (88); analysis of the raw data may determine whether children experiencing an episode of febrile malaria had a concurrent episode of diarrhoea. A meta-analysis of raw data sets from similar studies may help to provide a more robust answer to the question of whether malaria causes significant intestinal pathology.

Very few studies presented a breakdown of the prevalence of diarrhoea and malaria by age, parasite density, parasite species or severity of malaria, all of which may affect the strength of any association. Sodiende et al. found that parasite density did not predict the likelihood of concurrent diarrhoea (29) although, interestingly, the majority of incidents of malaria-associated diarrhoea were in individuals with relatively low parasite densities (<1,000 parasites/μl in 52% of patients) (29). Given this observation, it may also be worthwhile to explore the prevalence of diarrhoea in people with low or very low level malaria infections, including those with submicroscopic infections (detectable by PCR). Only three the studies in this review included infections detected by PCR, despite their clinical importance (89), raising the possibility—for the case control studies—that some of the “controls” may in fact have carried subclinical malaria infections.

Although diarrhoea was reported in conjunction with all of the human-infective malaria species, the majority of the studies (82%) related to *P. falciparum* infection. Five studies were able to stratify diarrhoea prevalence by *Plasmodium* species (*P. falciparum* vs. *P. vivax*); of these, one study found a significant 4-fold higher risk of diarrhoea among *P. falciparum* cases compared to *P. vivax* but this was not confirmed elsewhere (Supplementary Table 5). Future studies comparing the incidence of diarrhoea in association with different malaria species, especially comparing sequestering and non-sequestering parasites, may throw light on the mechanisms of any underlying intestinal pathology. Similarly, although anecdotal clinical data suggest a higher incidence of diarrhoea in malaria-infected adults compared to children, a suggestion that is supported by a superficial comparison of the studies included here, there are many potential confounders of this association that cannot be explored with the currently available data and it would unwise to draw any conclusions without further studies. In summary, there is currently insufficient evidence to determine whether parasite species or density, or the age of the subject, affects the relationship between malaria infection and diarrhoea.

Our systematic search identified very few studies of intestinal pathology in people with malaria infections, although we did not search explicitly for this term. Nevertheless, the earliest publication we were able to access, concerning a series of fatal malaria cases among British soldiers serving in Macedonia in 1916–1917, reported intestinal pathology in eleven of 50 cases (26). The most frequent observations were congestion of the blood-vessels, villous and submucosal hemorrhages, localized patches of necrosis and, in three cases, obstruction of intestinal capillaries by parasitized red blood cells. In other studies, *P. falciparum* infection has been associated with increased intestinal permeability (90) and parasites have been reported in the intestinal mucosa in human autopsy studies; this has been described as parasite sequestration (in villous capillaries) (24, 25, 91). These observations, although few in number, are consistent with observations from experimental studies in animal models (8, 9, 92) suggesting that more detailed investigations of human intestinal material may be worthwhile.

As there is a potential for bias in restricting the analysis to studies published in English we performed a search for articles not in English but with English abstracts or keywords and identified 305 hits (0 in PubMed, 26 in Web of Science, 82 in EMBASE/OVID, and 197 in CABI Global Health); the majority were reviews or conference abstracts (reflecting the nature of the CABI Global Health database). Only one article appeared relevant from the English abstract [a study of 61 children in Brazil of whom 34% were reported to have diarrhoea (93)], but a full-text review of the article (in Portuguese) was not performed. A search using foreign language terms may reveal additional papers (i.e., lacking English abstracts or keywords) but translation of such information was beyond the scope of this study.

In summary, this systematic review of the literature has revealed very diverse estimates of the prevalence of diarrhoea in malaria patients and *vice versa*; these estimates are based on a relatively small number of studies and interpretation of the data is hampered by numerous limitations including diverse demographics, imperfect methodologies, lack of clear definitions of diarrhoea, and lack of access to raw data. We conclude, therefore, that there is currently insufficient evidence to determine whether diarrhoea is a significant feature of malaria infection and thus whether malaria-associated intestinal pathology might underlie the increased risk of enteropathogenic bacteraemia associated with malaria. It is likely that a review of existing data sets from large community-based studies, including longitudinal cohort studies and randomized controlled trials of malaria interventions such as vaccines and insecticide treated bed-nets, could reveal such associations, if they exist, as such studies tend to adhere to rigorous standards of data collection and analysis including detailed demographic data and consistent case definitions. In support of this assertion, an initial examination of the publicly accessible database for the Global Enteric Multi-Center Study (GEMS) (a case-control study of acute diarrhoea in almost 10,000 children under 5 years of age) suggests that 25% of children with diarrhoea but only 8% of controls had concomitant malaria infections (https://clinepidb.org/ce/app/record/dataset/DS_841a9f5259). Alternatively, comprehensive longitudinal cohort studies, accompanied by detailed characterization of both the malaria infection (species, density, impact, and duration of infection) and any associated intestinal symptoms (frequency and consistency of stools, presence of blood, mucus, and pathogenic micro-organisms) are required to determine the clinical and biomedical importance of intestinal disease associated with malaria infections.

## Data Availability Statement

The raw data supporting the conclusions of this article will be made available by the authors, without undue reservation.

## Author Contributions

IS, ER, and JM: study concept and design. IS and JM: manuscript screening and data extraction. IS, AE, JM, and CB: analysis and statistical approach. IS, AE, CB, ER, and JM: drafting and revision of manuscript. All authors: approval for submission.

## Conflict of Interest

The authors declare that the research was conducted in the absence of any commercial or financial relationships that could be construed as a potential conflict of interest.

## References

[1] World Health Organization Guidelines for the Treatment of Malaria. World Health Organization (2015).

[2] Coban C Lee MSJ Ishii KJ. Tissue-specific immunopathology during malaria infection. Nat Rev Immunol. (2018) 18:266. 10.1038/nri.2017.138 29332936 PMC7097228

[3] Mabey DC Brown A Greenwood BM. *Plasmodium falciparum* malaria and Salmonella infections in Gambian children. J Infect Dis. (1987) 155:1319–21. 10.1093/infdis/155.6.1319 3553352

[4] Church J Maitland K. Invasive bacterial co-infection in African children with *Plasmodium falciparum* malaria: a systematic review. BMC Med. (2014) 12:31. 10.1186/1741-7015-12-31 24548672 PMC3928319

[5] Bronzan RN Taylor TE Mwenechanya J Tembo M Kayira K Bwanaisa L. Bacteremia in Malawian children with severe malaria: prevalence, etiology, HIV coinfection, and outcome. J Infect Dis. (2007) 195:895–904. 10.1086/511437 17299721

[6] Cunnington AJ de Souza JB Walther M Riley EM. Malaria impairs resistance to Salmonella through heme- and heme oxygenase-dependent dysfunctional granulocyte mobilization. Nat Med. (2012) 18:120–7. 10.1038/nm.2601 22179318 PMC3272454

[7] Lokken KL Mooney JP Butler BP Xavier MN Chau JY Schaltenberg N. Malaria parasite infection compromises control of concurrent systemic non-typhoidal Salmonella infection via IL-10-mediated alteration of myeloid cell function. PLoS Pathog. (2014) 10:e1004049. 10.1371/journal.ppat.1004049 24787713 PMC4006898

[8] Mooney JP Lokken KL Byndloss MX George MD Velazquez EM Faber F. Inflammation-associated alterations to the intestinal microbiota reduce colonization resistance against non-typhoidal Salmonella during concurrent malaria parasite infection. Sci Rep. (2015) 5:14603. 10.1038/srep14603 26434367 PMC4592952

[9] Alamer E Carpio VH Ibitokou SA Kirtley ML Phoenix IR Opata MM. Dissemination of non-typhoidal Salmonella during *Plasmodium chabaudi* infection affects anti-malarial immunity. Parasitol Res. (2019) 118:2277–85. 10.1007/s00436-019-06349-z 31119381 PMC6686885

[10] Chau JY Tiffany CM Nimishakavi S Lawrence JA Pakpour N Mooney JP. Malaria-associated L-arginine deficiency induces mast cell-associated disruption to intestinal barrier defenses against nontyphoidal Salmonella bacteremia. Infect Immunity. (2013) 81:3515–26. 10.1128/IAI.00380-13 23690397 PMC3811760

[11] Kaiser M Löwa A Ulrich M Ellerbrok H Goffe AS Blasse A. Wild chimpanzees infected with 5 Plasmodium species. Emerg Infect Dis. (2010) 16:1956–9. 10.3201/eid1612.100424 21122230 PMC3294549

[12] Abkallo HM Liu W Hokama S Ferreira PE Nakazawa S Maeno Y. DNA from pre-erythrocytic stage malaria parasites is detectable by PCR in the faeces and blood of hosts. Int J Parasitol. (2014) 44:467–73. 10.1016/j.ijpara.2014.03.002 24704779

[13] Campos SDE Pires JR Nascimento CL Dutra G Torres-Filho RA Toma HK Analysis of hematologic and serum chemistry values of Spheniscus magellanicus with molecular detection of avian malarial parasites (*Plasmodium* spp.). Pesquisa Veterinária Brasileira. (2014) 34:1236–42. 10.1590/S0100-736X2014001200016

[14] Greenberg AE Lobel HO. Mortality from *Plasmodium falciparum* malaria in travelers from the United States, 1959 to 1987. Ann Intern Med. (1990) 113:326–7. 10.7326/0003-4819-113-4-326 2197915

[15] Song HH O SO Kim SH Moon SH Kim JB Yoon JW. Clinical features of *Plasmodium vivax* malaria. Korean J Int Med. (2003) 18:220–4. 10.3904/kjim.2003.18.4.220 14717229 PMC4531638

[16] Bottieau E Clerinx J Van Den Enden E Van Esbroeck M Colebunders R Van Gompel A. Imported non-*Plasmodium falciparum* malaria: a five-year prospective study in a European referral center. Am J Trop Med Hyg. (2006) 75:133–8. 10.4269/ajtmh.2006.75.133 16837719

[17] Chung HC Wang JT Sun HY Wang JL Lo YC Sheng WH. Clinical experience of 17 cases of imported malaria at a Taiwan university hospital, 1999-2005. J Microbiol Immunol Infect. (2007) 40:209–15. 17639160

[18] Raposo CCBS Santos JB Santos GMCD Gonçalves EDGD da Silva RA. *Plasmodium vivax* malaria: related factors to severity in the State of Maranhão, Brazil. Revista da Sociedade Brasileira de Medicina Tropical. (2013) 46:67–72. 10.1590/0037-868212382013 23563828

[19] World Health Organization Malaria - Disease Information. World Health Organization (2011). Available: https://www.who.int/ith/diseases/malaria/en/ (accessed July 01, 2020).

[20] Camponovo F Bever CA Galactionova K Smith T Penny MA. Incidence and admission rates for severe malaria and their impact on mortality in Africa. Malaria J. (2017) 16:1. 10.1186/s12936-016-1650-6 28049519 PMC5209951

[21] Troeger C Blacker BF Khalil IA Rao PC Cao S Zimsen SRM Estimates of the global, regional, and national morbidity, mortality, and aetiologies of diarrhoea in 195 countries: a systematic analysis for the Global Burden of Disease Study 2016. Lancet Infect Dis. (2018) 18:1211–28. 10.1016/S1473-3099(18)30362-1 30243583 PMC6202444

[22] Masangwi S Ferguson N Grimason A Morse T Kazembe L. The pattern of variation between diarrhea and malaria coexistence with corresponding risk factors in, chikhwawa, malawi: a bivariate multilevel analysis. Int J Env Res Public Health. (2015) 12:8526–41. 10.3390/ijerph120708526 26197332 PMC4515734

[23] Mooney JP Butler BP Lokken KL Xavier MN Chau JY Schaltenberg N. The mucosal inflammatory response to non-typhoidal Salmonella in the intestine is blunted by IL-10 during concurrent malaria parasite infection. Mucosal Immunol. (2014) 7:1302–11. 10.1038/mi.2014.18 24670425 PMC4177018

[24] Pongponratn E Riganti M Punpoowong B Aikawa M. Microvascular sequestration of parasitized erythrocytes in human falciparum malaria: a pathological study. Am J Trop Med Hyg. (1991) 44:168–75. 10.4269/ajtmh.1991.44.168 2012260

[25] Seydel KB Milner DA Jr Kamiza SB Molyneux ME Taylor TE. The distribution and intensity of parasite sequestration in comatose Malawian children. J infect Dis. (2006) 194:208–15. 10.1086/505078 16779727 PMC1515074

[26] Dudgeon LS Clarke C An investigation on fatal cases of pernicious malaria caused by *Plasmodium falciparum* in Macedonia. QJM Int J Med. (1919) 12, 372–90. 10.1093/qjmed/os-12.48.372

[27] Prasad RN Virk KJ. Malaria as a cause of diarrhoea–a review. P N G Med J. (1993) 36:337–41. 10.1080/00357529.1993.9926566 7941765

[28] Bramer WM Rethlefsen ML Kleijnen J Franco OH. Optimal database combinations for literature searches in systematic reviews: a prospective exploratory study. Syst Rev. (2017) 6:245. 10.1186/s13643-017-0644-y 29208034 PMC5718002

[29] Sodeinde O Gbadegesin RA Ademowo OG Adeyemo AA. Lack of association between falciparum malaria parasitemia and acute diarrhea in Nigerian children. Am J Trop Med Hyg. (1997) 57:702–5. 10.4269/ajtmh.1997.57.702 9430531

[30] Olsson RA Johnston EH. Histopathologic changes and small-bowel absorption in falciparum malaria. Am J Trop Med Hyg. (1969) 18:355–9. 10.4269/ajtmh.1969.18.355 4889829

[31] Stein CM Gelfand M. The clinical features and laboratory findings in acute *Plasmodium falciparum* malaria in Harare, Zimbabwe. Cent Afr J Med. (1985) 31:166–70. 3910259

[32] Lepage P Bogaerts J Van Goethem C Ntahorutaba M Nsengumuremyi F Hitimana DG. Community-acquired bacteraemia in African children. Lancet. (1987) 1:1458–61. 10.1016/S0140-6736(87)92207-0 2885453

[33] Müller O Moser R. The clinical and parasitological presentation of *Plasmodium falciparum* malaria in Uganda is unaffected by HIV-1 infection. Trans R Soc Trop Med Hyg. (1990) 84:336–8. 10.1016/0035-9203(90)90306-Y 2260160

[34] Ibhanesebhor SE. Clinical characteristics of neonatal malaria. J Trop Pediatr. (1995) 41:330–3. 10.1093/tropej/41.6.330 8606438

[35] Sodeinde O Adeyemo AA Gbadegesin RA Olaleye BO Ajayi-Obe KE Ademowo OG. Interaction between acute diarrhoea and falciparum malaria in Nigerian children. J Diarrhoeal Dis Res. (1996) 14:269–73. 9203790

[36] Sheiban AK. Prognosis of malaria associated severe acute renal failure in children. Renal Failure. (1999) 21:63–6. 10.3109/08860229909066970 10048118

[37] Sowunmi A Ogundahunsi OAT Falade CO Gbotosho GO Oduola AMJ. Gastrointestinal manifestations of acute falciparum malaria in children. Acta Trop. (2000) 74:73–6. 10.1016/S0001-706X(99)00043-1 10643910

[38] Hozhabri S Akhtar S Rahbar MH Luby SP. Prevalence of plasmodium slide positivity among the children treated for malaria, Jhangara, Sindh. J Pak Med Assoc. (2000) 50:401–5. 11191438

[39] Singh B Kim Sung L Matusop A Radhakrishnan A Shamsul SS Cox-Singh J. A large focus of naturally acquired *Plasmodium knowlesi* infections in human beings. Lancet. (2004) 363:1017–24. 10.1016/S0140-6736(04)15836-4 15051281

[40] Khan MA Mekan SF Abbas Z Smego RA Jr. Concurrent malaria and enteric fever in Pakistan. Singapore Med J. (2005) 46:635–8. 16228096

[41] Fryauff DJ Owusu-Agyei S Utz G Baird JK Koram KA Binka F. Mefloquine treatment for uncomplicated falciparum malaria in young children 6-24 months of age in northern Ghana. Am J Trop Med Hyg. (2007) 76:224–31. 10.4269/ajtmh.2007.76.224 17297028

[42] Ansari S Khoharo HK Abro A Akhund IA Qureshi F. Thrombocytopenia in *plasmodium falciparum* malaria. J Ayub Med Coll Abbottabad. (2009) 21:145–7. 20524493

[43] Rasheed A Saeed S Khan SA. Clinical and laboratory findings in acute malaria caused by various plasmodium species. J Pak Med Assoc. (2009) 59:220–3. 19402282

[44] Nanda NC Rath P Acharya J Mishra P Mishra SK. Falciparum malaria in children—a brief report of 305 patients from Rourkela, Eastern India. Indian J Pediatr. (2011) 78:475–7. 10.1007/s12098-010-0287-7 21088935

[45] Memon IA Tariq S Jamil A Prevalence of malaria in young febrile children. Pakistan Paediatr J. (2012) 36:70–4.

[46] Ketema T Bacha K. Plasmodium vivax associated severe malaria complications among children in some malaria endemic areas of Ethiopia. BMC Public Health. (2013) 13:637. 10.1186/1471-2458-13-637 23834734 PMC3724694

[47] Kamal K Mode of presentation and susceptibility to treatment of malaria in children at thal, a remote area of KP, Pakistan. Ann Pak Inst Med Sci. (2013) 9:74–7.

[48] Arnold BJ Tangpukdee N Krudsood S Wilairatana P. Risk factors of shock in severe falciparum malaria. Southeast Asian J Trop Med Public Health. (2013) 44:541–50. 24050086

[49] He D Zhang Y Liu X Guo S Zhao D Zhu Y. Epidemiological and clinical features of *Plasmodium falciparum* malaria in united nations personnel in Western Bahr el Ghazal State, South Sudan. PLoS ONE. (2013) 8:e55220. 10.1371/journal.pone.0055220 23372839 PMC3555950

[50] Tao ZY Fang Q Liu X Culleton R Tao L Xia H. Congenital Malaria in China. PLOS Negl Trop Dis. (2014) 8:e2622. 10.1371/journal.pntd.0002622 24626148 PMC3953009

[51] Dotrário AB Menon LJB Bollela VR Martinez R de Almeida e Araújo DC da Fonseca BAL Malaria and other febrile diseases among travellers: the experience of a reference centre located outside the Brazilian Amazon Region. Malaria J. (2016) 15:294 10.1186/s12936-016-1347-x PMC4882771 27230739

[52] Irawati N Kurniawan B Suwandi JF Hasmiwati Tjong DH Kanedi M Determination of the falciparum malaria resistance to artemisinin-based combination therapies in Pesawaran, Lampung, Indonesia. Asian J Epidemiol. (2017) 10:19–25. 10.3923/aje.2017.19.25

[53] Nateghpour M Hosseininasab A Farrokhnia M Dastouri F Alidoosti K Sadequi D. Species-dependent clinical findings of malaria caused by various plasmodia in an endemic area of Kerman Province, Southeastern Iran. Iran J Public Health. (2017) 46:525–9. 28540269 PMC5439042

[54] O'Holohan DR. Clinical and laboratory presentation of malaria: an analysis of one thousand subjects with malaria parasitaemia. J Trop Med Hyg. (1976) 79:191–6. 794512

[55] Kean BH Reilly PCJr. Malaria—the mime: Recent lessons from a group of civilian travellers. Am J Med. (1976) 61:159–64. 10.1016/0002-9343(76)90164-9 782238

[56] Gordon S Brennessel DJ Goldstein JA Rosner F. *Malaria*. A city hospital experience. Arch Int Med. (1988) 148:1569–71. 10.1001/archinte.1988.00380070071017 3289521

[57] Jelinek T Nothdurft HD Löscher T. Malaria in nonimmune travelers: a synopsis of history, symptoms, and treatment in 160 patients. J Travel Med. (2006) 1:199–202. 10.1111/j.1708-8305.1994.tb00595.x 9815339

[58] Robinson P Jenney AW Tachado M Yung A Manitta J Taylor K. Imported malaria treated in Melbourne, Australia: epidemiology and clinical features in 246 patients. J Travel Med. (2001) 8:76–81. 10.2310/7060.2001.24309 11285166

[59] Chalumeau M Holvoet L Cheron G Minodier P Foix-L'Helias L Ovetchkine P. Delay in diagnosis of imported *Plasmodium falciparum* malaria in children. Eur J Clin Microbiol Infect Dis. (2006) 25:186–9. 10.1007/s10096-006-0105-3 16525777

[60] Zaher T Ahmadi M Ibrahim A El-Bahnasawy M Gouda H Shahat SA. Malaria in Egypt, Saudi Arabia and Yemen: a clinical pilot study. J Egypt Soc Parasitol. (2007) 37:969–76. 18383796

[61] Ansart S Perez L Thellier M Danis M Bricaire F Caumes E. Predictive factors of imported malaria in 272 febrile returning travelers seen as outpatients. J Travel Med. (2010) 17:124–9. 10.1111/j.1708-8305.2009.00382.x 20412180

[62] Govardhini P Manoharan A Subramanian S Mohapatra SS Jambulingam P Das PK. Symptomatic diagnosis of *Plasmodium falciparum* malaria in field conditions. Indian J Malariol. (1991) 28:55–62. 1915984

[63] Laurentz R Manoempil PS. Malaria diarrhoea in children. Asia Pacific J Public Health. (1995) 8:186–9. 10.1177/101053959500800308 10050187

[64] Sodemann M Jakobsen MS Mølbak K Alvarenga IC Martins C Aaby P. Malaria parasitemia and childhood diarrhea in a peri-urban area of Guinea-Bissau. Am J Trop Med Hyg. (1999) 61:336–8. 10.4269/ajtmh.1999.61.336 10463690

[65] Ashie GK Mutocheluh M Owusu M Kwofie TB Akonor S Narkwa PW. Microbial pathogens associated with acute childhood diarrhoea in Kumasi, Ghana. BMC ResNotes. (2017) 10:264. 10.1186/s13104-017-2578-9 28693616 PMC5504554

[66] Lee LA Dogore R Redd SC Dogore E Metchock B Diabate J. Severe illness in African children with diarrhoea: implications for case management strategies. Bull World Health Organ. (1995) 73:779–85. 8907771 PMC2486685

[67] Ibadin OM Airauhi L Omoigberale AI Abiodun PO. Association of malarial parasitaemia with dehydrating diarrhoea in Nigerian children. J Health Popul Nutr. (2000) 18:115–8. 11057068

[68] Reither K Ignatius R Weitzel T Seidu-Korkor A Anyidoho L Saad E. Acute childhood diarrhoea in northern Ghana: epidemiological, clinical and microbiological characteristics. BMC Infect Dis. (2007) 7:104. 10.1186/1471-2334-7-104 17822541 PMC2018704

[69] Deogratias A-P Mushi MF Paterno L Tappe D Seni J Kabymera R. Prevalence and determinants of Campylobacter infection among under five children with acute watery diarrhea in Mwanza, North Tanzania. Arch Public Health. (2014) 72:17. 10.1186/2049-3258-72-17 24932408 PMC4057571

[70] Bonghaseh TD Ekaney DSM Budzi M Ekwen G Kyota S. Sub-acute intestinal obstruction – a rare complication of *Plasmodium falciparum* malaria in an adult: a case report. Journal of Medical Case Reports. (2018) 12:190. 10.1186/s13256-018-1730-z 29966528 PMC6029377

[71] Bin Mohanna MA Leishmaniasis, malaria, and schistosomiasis concurrently in an 8-year-old boy. Saudi Med J. (2015) 36:494–6. 10.15537/smj.2015.4.10757 25828290 PMC4404487

[72] Diallo MA Kane BS Ndiaye M Dieng M Diongue K Badiane AS. *Plasmodium falciparum* malaria co-infection with tick-borne relapsing fever in Dakar. Malaria J. (2017) 16:24. 10.1186/s12936-017-1682-6 28077149 PMC5225580

[73] Chowdhury F Chisti MJ Khan AH Chowdhury MA Pietroni MA. Salmonella Typhi and *Plasmodium falciparum* co-infection in a 12-year old girl with haemoglobin E trait from a non-malarious area in Bangladesh. J Health Popul Nutr. (2010) 28:529–31. 10.3329/jhpn.v28i5.6162 20941905 PMC2963776

[74] Cox-Singh J Davis TME Lee K-S Shamsul SSG Matusop A Ratnam S. *Plasmodium knowlesi* malaria in humans is widely distributed and potentially life threatening. Clin Infect Dis. (2008) 46:165–71. 10.1086/524888 18171245 PMC2533694

[75] Enger A Strand OA Ranheim T Hellum KB. Exflagellation of microgametocytes in *Plasmodium vivax* malaria: a diagnostic conundrum. Med Princ Pract. (2004) 13:298–300. 10.1159/000079533 15316267

[76] Christova I Petrov A Papa A Vutchev D Kalvatchev N Vatev N. Fatal outcome of coinfection of Crimean-Congo hemorrhagic fever and malaria. Jpn J Infect Dis. (2015) 68:131–4. 10.7883/yoken.JJID.2014.106 25420643

[77] Hussain WM Syed Zahid B Mohammad Ibrahim F Talal Mohammad K Tariq Ahmed M Samar B. Misdiagnosis of an imported case of malaria caused by *Plasmodium falciparum*. J Infect Dev Ctries. (2009) 3:112–4. 10.3855/jidc.58 19755740

[78] Nissen T Wynn R. The clinical case report: a review of its merits and limitations. BMC Res Notes. (2014) 7:264. 10.1186/1756-0500-7-264 24758689 PMC4001358

[79] Rogier C Ly AB Tall A Cissé B Trape JF. *Plasmodium falciparum* clinical malaria in Dielmo, a holoendemic area in Senegal: no influence of acquired immunity on initial symptomatology and severity of malaria attacks. Am J Trop Med Hyg. (1999) 60:410–20. 10.4269/ajtmh.1999.60.410 10466970

[80] Branch O Casapia WM Gamboa DV Hernandez JN Alava FF Roncal N. Clustered local transmission and asymptomatic *Plasmodium falciparum* and Plasmodium vivax malaria infections in a recently emerged, hypoendemic Peruvian Amazon community. Malar J. (2005) 4:27. 10.1186/1475-2875-4-27 15975146 PMC1190209

[81] Martins AC Araújo FM Braga CB Guimarães MGS Nogueira R Arruda RA. Clustering symptoms of non-severe malaria in semi-immune Amazonian patients. PeerJ. (2015) 3:e1325. 10.7717/peerj.1325 26500831 PMC4614890

[82] Akowuah E Nsiah K The pattern of malaria infection in and around the Kwame Nkrumah University of Science and Technology (KNUST) campus. Acta Sci Med Sci. (2019) 3:132–7.

[83] Anokye R Acheampong E Owusu I Isaac Obeng E Time series analysis of malaria in Kumasi: Using ARIMA models to forecast future incidence. Cogent Soc Sci. (2018) 4:1461544 10.1080/23311886.2018.1461544

[84] Wenzl HH Fine KD Schiller LR Fordtran JS. Determinants of decreased fecal consistency in patients with diarrhea. Gastroenterology. (1995) 108:1729–38. 10.1016/0016-5085(95)90134-5 7768377

[85] Akinbami FO Okereke JO Orimadegun AE. The bowel habits of adolescents in Nigeria. Trop Gastroenterol. (2011) 31:295–302. 21568146

[86] Panigrahi MK Kar SK Singh SP Ghoshal UC. Defecation frequency and stool form in a coastal eastern Indian population. J Neurogastroenterol Motility. (2013) 19:374–80. 10.5056/jnm.2013.19.3.374 23875105 PMC3714416

[87] Vandeputte D Falony G Vieira-Silva S Tito RY Joossens M Raes J. Stool consistency is strongly associated with gut microbiota richness and composition, enterotypes and bacterial growth rates. Gut. (2016) 65:57–62. 10.1136/gutjnl-2015-309618 26069274 PMC4717365

[88] Greenwood BM Byass P Greenwood AM Hayes RJ Menon A Shenton FC. Lack of an association between acute gastroenteritis, acute respiratory infections and malaria in young Gambian children. Transac R Soc Trop Med Hyg. (1989) 83:595–8. 10.1016/0035-9203(89)90364-7 2559509

[89] Chen I Clarke SE Gosling R Hamainza B Killeen G Magill A. “Asymptomatic” malaria: a chronic and debilitating infection that should be treated. PLoS Med. (2016) 13:e1001942. 10.1371/journal.pmed.1001942 26783752 PMC4718522

[90] Weinberg W Wilairatana P Meddings JB Ho M Vannaphan S Looareesuwan S. Increased gastrointestinal permeability in patients with *Plasmodium falciparum* malaria. Clin Infect Dis. (1997) 24:430–5. 10.1093/clinids/24.3.430 9114195

[91] Milner DAJr Lee JJ Frantzreb C Whitten RO Kamiza S Carr RA. Quantitative assessment of multiorgan sequestration of parasites in fatal pediatric cerebral malaria. J Infect Dis. (2015) 212:1317–21. 10.1093/infdis/jiv205 25852120 PMC4577044

[92] Denny JE Powers JB Castro HF Zhang J Joshi-Barve S Campagna SR. Differential sensitivity to plasmodium yoelii infection in C57BL/6 mice impacts gut-liver axis homeostasis. Sci Rep. (2019) 9:1–15. 10.1038/s41598-019-40266-6 30837607 PMC6401097

[93] Noronha E Alecrim MDGC Romero GAS Macêdo V. Estudo clínico da malária falciparum em crianças em Manaus, AM, Brasil. Revista da Sociedade Brasileira de Medicina Tropical. (2000) 33:185–90. 10.1590/S0037-86822000000200005 10881132

