# Supplementary material for: Does Malaria Cause Diarrhoea? A Systematic Review
Source: Front Med (Lausanne). 2020 Nov 19;7:589379. doi: 10.3389/fmed.2020.589379 (PMC7717985; doi:10.3389/fmed.2020.589379)
Supplement: Supplementary file 1 [file Data_Sheet_1.docx]

**Supplementary Digital Content:**

**Does Malaria cause Diarrhoea? A systematic review.**

Isatou Sey^1^; Ajoke M. Ehimiyein^1,2^; Christian Bottomley^3^; Eleanor M. Riley^1^; Jason P. Mooney^1^

^1^ Institute of Immunology and Infection Research, School of Biological Sciences, University of Edinburgh, Edinburgh, United Kingdom.

^2^ Department of Veterinary Medicine, Ahmadu Bello University, Zaria, Nigeria.

^3^ Department of Infectious Disease Epidemiology, London School of Hygiene and Tropical Medicine, London, United Kingdom.

Correspondence address: Jason P Mooney PhD, Institute of Immunology and Infection Research, School of Biological Sciences, Ashworth Laboratories, Kings Buildings, Charlotte Auerbach Rd, Edinburgh, EH9 3FL, UK

E-mail: [jason.mooney@ed.ac.uk](mailto:jason.mooney@ed.ac.uk)

**Summary of Supplementary Content:**

Table S1: 50 articles, chosen at random, which were excluded during review of their title and abstract, with reasons given.

Table S2: Summary of reasons for exclusion of articles following full-text review.

Table S3: Case reports of diarrhoea in patients from malaria endemic areas.

Table S4: Case reports of diarrhoea in patients having visited malaria endemic areas.

Table S5: Odds ratio of presenting with *P. falciparum* associated diarrhoea, compared to *P. vivax*.

List of Supplementary References

**Supplementary Tables:**

Table S1: 50 articles, chosen at random, which were excluded during review of their title and abstract, with reasons given.

| **Article** | **Reason for exclusion** | **Article** | **Reason for exclusion** |
| --- | --- | --- | --- |
| Adjuik *et al.,* 2015 (Adjuik, 2015) | Drug trial | Mahgoub *et al.,* 2012 (Mahgoub and Adam, 2012) | Malnutrition mortality (causes) |
| Anyanwu *et al.,* 2015 (Anyanwu et al., 2015) | Medicinal plants and what they treat | Nabongo *et al.,* 2014 (Nabongo et al., 2014) | Causes of childhood mortality |
| Bavinger *et al.,* 2017 (Bavinger et al., 2017) | Examples of global mortality | Nasveld *et al.,* 2005 (Nasveld and Kitchener, 2005) | Treatment of malaria |
| Bouth *et al.,* 2002 (Bouth-Denis et al., 2002) | Drug trial | Oberstaller *et al.,* 2014 (Oberstaller et al., 2014) | Cryptosporidium |
| Challand *et al.,* 2009 (Challand, 2009) | Drug trial | Oliveira *et al.,* 2005 (Oliveira et al., 2005) | Vaccine trial |
| Chinsembu *et al.,* 2016 (Chinsembu, 2016) | Medicinal plants for HIV treatment | Pattanayak *et al.,* 2009 (Pattanayak and Pfaff, 2009) | Behavior and health |
| Das *et al.,*1996 (Das et al., 1996) | Drug trial | Pilsczek *et al.,* 2015 (Pilsczek, 2015) | Respiratory infections |
| Dejon-Agobe *et al.,* 2019 (Dejon-Agobe et al., 2018) | Vaccine trial | Raj *et al.,* 2012 (Raj et al., 2012) | Medicinal plants |
| El Jaoudi *et al.,* 2010 (El Jaoudi et al., 2010) | Adverse reaction to mefloquine | Ramesh *et al.,* 2019 (Ramesh et al., 2019) | Paediatric illness, separate |
| Esamai *et al.,* 2005 (Esamai et al., 2005) | Drug trial | Salako *et al.,* 1990 (Salako et al., 1990) | Antimalarial treatment |
| Fanello *et al.,* 2008 (Fanello et al., 2008) | Drug trial | Savini *et al.,* 2013 (Savini et al., 2013) | Travel associated disease |
| Ferrer *et al.,* 2017 (Ferrer et al., 2017) | Community based health analysis | Sesay *et al.,* 2017 (Sesay et al., 2017) | Ebola |
| Gavazzi *et al.,* 2004 (Gavazzi et al., 2004) | Aging and infectious disease | Tavul *et al.,* 2018 (Tavul et al., 2018) | Drug trial |
| Goel *et al.,* 2007 (Goel et al., 2007) | Emerging infections | Tedbabe *et al.,* 2009 (Tedbabe et al., 2010) | Diarrhoea and malaria separate |
| Haque *et al.,* 2011 (Haque et al., 2011) | Factors relating to malaria risk | Thompson *et al.,* 2012 (Thompson et al., 2012) | Climate change and health |
| Hazel *et al.,* 2016 (Hazel et al., 2016) | Community health strategy | Tjitra *et al.,* 1995 (Tjitra et al., 1995) | Drug trial |
| Ibrahim *et al.,* 2016 (Ibrahim et al., 2016) | Drug trial in animals | Ursing *et al.,* 2016 (Ursing et al., 2016) | Drug trial |
| Ishengoma *et al.,* 2019 (Ishengoma et al., 2019) | Drug trial | Uzma Khalid *et al.,* 2013 (Uzma et al., 2013) | Malnutrition |
| Jande *et al.,* 2012 (Jande et al., 2012) | Drug prescribing in a hospital | Valech *et al.,* 2010 (Valecha et al., 2010) | Drug trial |
| Jiwani *et al.,* 2014 (Jiwani et al., 2014) | Disease prevention campaign | Vilkman *et al.,* 2016 (Vilkman et al., 2016) | Travellers health and behaviour |
| Kahabuka *et al.,* 2012 (Kahabuka et al., 2013) | Diarrhoea and malaria separate | Waldman *et al.,* 2018 (Waldman and Balskus, 2018) | Microbiota |
| Kam *et al.,* 2016 (Kam (van der) et al., 2016) | Nutrition trial | Watt *et al.,* 1994 (Watt et al., 1994) | Drug trial |
| Lainez *et al.,* 2012 (Laínez et al., 2012) | Community health program | Xu *et al.,* 2004 (Xu et al., 2004) | Cryptosporidium |
| Lee *et al.,* 2019 (Lee and Ryu, 2019) | Literature review | Yakoob *et al.,* 2011 (Yakoob et al., 2011) | Zinc supplementation |
| Macete *et al.,* 2006 (Macete et al., 2006) | Drug trial | Zoungrana *et al.,* 2008 (Zoungrana et al., 2008) | Drug trial |

Table S2: Summary of reasons for exclusion of 133 articles following full-text review of ‘plasmodium’ and ‘diarrhea’ results.

| **Reason for exclusion** | **Number of papers excluded, (%)** |
| --- | --- |
| Malaria and Diarrhoea are separate issue, not associated. | 28 (21%) |
| No appropriate malaria diagnosis *(must be microscopy, RDT or PCR confirmed)* | 17 (13%) |
| Drug adverse event or trial with no control group | 16 (12%) |
| No diarrhoea actually reported | 14 (11%) |
| Article not in English, only abstract is | 12 (9%) |
| Study design not appropriate | 13 (10%) |
| Full text unavailable for review | 10 (8%) |
| Review article | 8 (6%) |
| Diarrhoea not distinguished from other gastrointestinal symptoms (e.g. diarrhoea/vomiting) | 6 (5%) |
| No malaria found | 6 (5%) |
| Duplicate article | 3 (2%) |

Table S3: Case reports of diarrhoea in patients from malaria endemic areas.

| **Author** | **Origin** | **Patient(s)** | **Malaria parasite diagnosis** | | **Comorbidities** | **History of ‘Diarrhoea’** |
| --- | --- | --- | --- | --- | --- | --- |
|  |  |  | **Method** | **Species** |  |  |
| Chinnan et al, 2005 (Chinnan and Mishra, 2005) | India | 1F, 12 years old | Microscopy | *P. falciparum* | none | 2-day history of diarrhoea prior to presentation at hospital |
| Cox-Singh et al, 2008 (Cox-Singh et al., 2008) | Malaysia | 1M 69 years old, 1F 66 years old | Microscopy | *P. malariae* | none | Both had a 3-day history of diarrhoea prior to presentation at hospital, ‘Case 1’ and ‘Case 2’ |
| Chowdhury et al, 2010 (Chowdhury et al., 2010) | Bangladesh | 1F, 12 years old | Microscopy | *P. falciparum* | *S*. Typhi | 23-day history of diarrhoea prior to presentation at hospital |
| Vinod et al, 2012 (Vinod et al., 2012) | India | 1M, 21 years old | Microscopy | *P. vivax* | None (stool & blood culture negative) | Patient 1: 2 day history of loose water stool (8-10 episodes/day, no blood, mucus or foul smell). |
| Bin Mohanna et al, 2015 (Bin Mohanna, 2015) | Yemen | 1M, 8 years old | Microscopy | *P. falciparum* | Leishmania & Schistosomiasis | 4- week history of intermittent diarrhoea prior to presentation at hospital |
| Diallo et al, 2017 (Diallo et al., 2017) | Senegal | 1M, 28 years old | Microscopy | *P. falciparum* | Borreliosis | Diarrhoea lasted 3 days |
| Bonghaseh et al, 2018 (Bonghaseh et al., 2018) | Cameroon | 1M, 42 years old | RDT | *P. falciparum* | none | 4 day history of NO BOWEL MOVEMENT |

Note: Male (M) and Female (F).

Table S4: Case reports of diarrhoea in patients having visited malaria endemic areas.

| **Author** | **Country of Infection** | **Patient(s)** | **Malaria parasite diagnosis** | | | **Comorbidities** | **History of ‘Diarrhoea’** |
| --- | --- | --- | --- | --- | --- | --- | --- |
|  |  |  | **Country of Diagnosis** | **Method** | **Species** |  |  |
| de Caprariis, 1984 (de Caprariis and Giron, 1984) | Pakistan | 1F, 60 years old | USA | Microscopy | *P. vivax* | none | 14-day history of diarrhoea prior to presentation at hospital |
| Klingelberger, 1986 (Klingelberger, 1989) | The Philippines | 1M, 27 years old | USA | Microscopy | *P. vivax* | none | 7-day history of diarrhoea prior to presentation at hospital. Diarrhoea abated on the 5^th^ day of illness. |
| Miller et al, 1994 (Miller et al., 1994) | Kenya | 1M, 36 years old | UK | Microscopy | *P. falciparum* | none | 2 day history of diarrhoea prior to presentation at hospital, case ‘4’ |
| Iftikhar et al, 1995 (Iftikhar and Roistacher, 1995) | Unclear, possible import into USA | 1F, 22 years old | USA | Microscopy | *P. falciparum* | none | 10 day history of water, non-bloody diarrhoea |
| Enger et al, 2004 (Enger et al., 2004) | Ethiopia | 1M, 37 years old | Norway | Microscopy | *P. vivax* | HIV and Isospora belli | 4-MONTH history of diarrhoea prior to presentation at hospital |
| Uchiyama et al, 2004 (Uchiyama et al., 2004) | Guinea | 1M, 22 years old | Japan | Microscopy | *P. falciparum* | none | 4-day history of diarrhoea (no blood) prior to presentation at hospital |
| Maguire et al, 2007 (Maguire et al., 2007) | Afghanistan | 1M, 21 years old | USA | Microscopy | *P. vivax* | none | 5-day history of diarrhoea prior to presentation at hospital |
| Hussain et al, 2009 (Hussain et al., 2009) | Pakistan | 1M, 46 years old | Saudi Arabia | Microscopy | *P. falciparum* | *none* | 1-day history of bloody diarrhoea prior to presentation at hospital |
| De Laval et al, 2010 (de Laval et al., 2010) | Cote d’Ivoire | 6M, 30-36 years old | France | Microscopy | *P. ovale* | none | 2 of the six cases reported abdominal pain and diarrhoea, data unclear on who |
| Johnson et al, 2013 (Johnson et al., 2013) | Democratic Republic of Congo | 1M, 3 years old | USA | Microscopy | *P. ovale* | none | ‘recent’ travel, presented with abdominal pain, vomiting and diarrhoea. |
| Cordina et al, 2014 (Cordina et al., 2014) | Borneo | 1F, 33 years old | UK | PCR and Microscopy | *P. knowlesi* | none | 2-day history of diarrhoea prior to presentation at hospital, persisting for 13 |
| Choi et al, 2016 (Choi et al., 2016) | South Africa, Kenya, and Nigeria | 1M, 15 years old | Korea | Microscopy | *P. falciparum* | none | 5-day history of diarrhoea & abdominal pain prior to presentation at hospital |
| Christova et al, 2015 (Christova et al., 2015) | Zambia | 1M, 39 years old | Bulgaria | Microscopy | *P. falciparum* | Crimean-Congo Haemorrhagic Fever | Onset of bloody diarrhoea 2 days after admission to hospital |
| Anani et al, 2017 (Anani et al., 2017) | Kenya | 1F, 84 years old | USA | Microscopy | *P. falciparum* | none | 3-day history of intermittent diarrhoea prior to presentation at hospital |
| Malchrzak et al, 2018 (Malchrzak et al., 2018) | Nigeria | 1M, 35 years old | Poland | Microscopy | *P. falciparum* | IBS & HBV | Diarrhoea occurred in hospital, no details. |

Note: Male (M) and Female (F).

Table S5: Odds ratio of presenting with *P. falciparum* associated diarrhoea, compared to *P. vivax*.

| **Author** | ***P. falciparum*** | | ***P. vivax*** | | **Odds Ratio (95% CI),**  **p value** |
| --- | --- | --- | --- | --- | --- |
|  | **Number, n** | **Diarrhoea positive, n (%)** | **Number, n** | **Diarrhoea positive, n (%)** |  |
| Robinson et al, 2001 | 71 | 21 (29.6) | 264 | 25 (9.5) | 4.0 (2.1-7.7), p<0.0001 |
| Rasheed et al, | 311 | 14 (4.5) | 100 | 2 (2.0) | 2.3 (0.5-10.3), p=0.26 |
| Branch et al, | 62 | 4 (6.5) | 212 | 19 (9.0) | 0.7 (0.2-2.1), p=0.53 |
| O’Holohan et al, | 429 | 21 (4.9) | 848 | 34 (4.0) | 1.2 (0.7-2.2), p=0.46 |
| Nateghpour et al, 2017 | 10 | 0 (0) | 314 | 28 (8.9) | 0.0 (0-4.0), p=1.00 |

**Supplementary References:**

Adjuik, M.A.A.R.A.A.R.A.E.A.B.M.S.B.H.B.K.I.B.Q.B.E. (2015). The effect of dosing strategies on the therapeutic efficacy of artesunate-amodiaquine for uncomplicated malaria: a meta-analysis of individual patient data. *BMC Medicine* 13(1)**,** 66. doi: 10.1186/s12916-015-0301-z.

Anani, W.Q., Smith, G.P., Irani, M., and Puca, K.E. (2017). A report of cerebral malaria treated with automated red blood cell exchange. *Transfusion* 57(4)**,** 985-988. doi: 10.1111/trf.14013.

Anyanwu, G.O., Nisar ur, R., Onyeneke, C.E., and Rauf, K. (2015). Medicinal plants of the genus Anthocleista—A review of their ethnobotany, phytochemistry and pharmacology. *Journal of Ethnopharmacology* 175**,** 648-667. doi: <https://doi.org/10.1016/j.jep.2015.09.032>.

Bavinger, J.C., Wise, P., and Bendavid, E. (2017). The relationship between burden of childhood disease and foreign aid for child health. *BMC Health Services Research* 17(1)**,** 655. doi: 10.1186/s12913-017-2540-5.

Bin Mohanna, M.A. (2015). Leishmaniasis, malaria, and schistosomiasis concurrently in an 8-year-old boy. *Saudi Med J* 36(4)**,** 494-496. doi: 10.15537/smj.2015.4.10757.

Bonghaseh, T.D., Ekaney, D.S.M., Budzi, M., Ekwen, G., and Kyota, S. (2018). Sub-acute intestinal obstruction – a rare complication of Plasmodium falciparum malaria in an adult: a case report. *Journal of Medical Case Reports* 12(1)**,** 190. doi: 10.1186/s13256-018-1730-z.

Bouth-Denis, M., Davis, T.M.E., Hewitt, S., Incardona, S., Nimol, K., Fandeur, T., et al. (2002). Efficacy and Safety of Dihydroartemisinin-Piperaquine (Artekin) in Cambodian Children and Adults with Uncomplicated Falciparum Malaria. *Clinical Infectious Diseases* 35(12)**,** 1469-1476. doi: 10.1086/344647.

Challand, S.W.M. (2009). A Clinical Trial of the Traditional Medicine Vernonia amygdalina in the Treatment of Uncomplicated Malaria. *The Journal of Alternative and Complementary Medicine* 15(11)**,** 1231-1237. doi: 10.1089/acm.2009.0098.

Chinnan, N.K., and Mishra, P. (2005). Fever and shock in a child: How 'good' is a good blood test? *The Canadian journal of infectious diseases & medical microbiology = Journal canadien des maladies infectieuses et de la microbiologie medicale* 16(5)**,** 301-303. doi: 10.1155/2005/404983.

Chinsembu, K.C. (2016). Ethnobotanical Study of Plants Used in the Management of HIV/AIDS-Related Diseases in Livingstone, Southern Province, Zambia. *Evidence-Based Complementary and Alternative Medicine* 2016**,** 4238625. doi: 10.1155/2016/4238625.

Choi, I.H., Hwang, P.H., Choi, S.I., Lee, D.Y., and Kim, M.S. (2016). Delayed Diagnosis of Falciparum Malaria with Acute Kidney Injury. *J Korean Med Sci* 31(9)**,** 1499-1502. doi: 10.3346/jkms.2016.31.9.1499.

Chowdhury, F., Chisti, M.J., Khan, A.H., Chowdhury, M.A., and Pietroni, M.A. (2010). Salmonella Typhi and Plasmodium falciparum co-infection in a 12-year old girl with haemoglobin E trait from a non-malarious area in Bangladesh. *J Health Popul Nutr* 28(5)**,** 529-531. doi: 10.3329/jhpn.v28i5.6162.

Christova, I., Petrov, A., Papa, A., Vutchev, D., Kalvatchev, N., Vatev, N., et al. (2015). Fatal outcome of coinfection of Crimean-Congo hemorrhagic fever and malaria. *Jpn J Infect Dis* 68(2)**,** 131-134. doi: 10.7883/yoken.JJID.2014.106.

Cordina, C.J., Culleton, R., Jones, B.L., Smith, C.C., MacConnachie, A.A., Coyne, M.J., et al. (2014). Plasmodium knowlesi: Clinical Presentation and Laboratory Diagnosis of the First Human Case in a Scottish Traveler. *Journal of Travel Medicine* 21(5)**,** 357-360. doi: 10.1111/jtm.12131.

Cox-Singh, J., Davis, T.M.E., Lee, K.-S., Shamsul, S.S.G., Matusop, A., Ratnam, S., et al. (2008). Plasmodium knowlesi malaria in humans is widely distributed and potentially life threatening. *Clinical infectious diseases : an official publication of the Infectious Diseases Society of America* 46(2)**,** 165-171. doi: 10.1086/524888.

Das, B.S., Thurnham, D.I., and Das, D.B. (1996). Plasma alpha-tocopherol, retinol, and carotenoids in children with falciparum malaria. *Am J Clin Nutr* 64(1)**,** 94-100. doi: 10.1093/ajcn/64.1.94.

de Caprariis, P.J., and Giron, J.A. (1984). A Pakistani woman who arrived with diarrhea. *Hosp Pract (Off Ed)* 19(9)**,** 134d-134e.

de Laval, F., Oliver, M., Rapp, C., Pommier de Santi, V., Mendibil, A., Deparis, X., et al. (2010). The challenge of diagnosing Plasmodium ovale malaria in travellers: report of six clustered cases in french soldiers returning from West Africa. *Malaria Journal* 9(1)**,** 358. doi: 10.1186/1475-2875-9-358.

Dejon-Agobe, J.C., Ateba-Ngoa, U., Lalremruata, A., Homoet, A., Engelhorn, J., Nouatin, O.P., et al. (2018). Controlled Human Malaria Infection of Healthy Adults With Lifelong Malaria Exposure to Assess Safety, Immunogenicity, and Efficacy of the Asexual Blood Stage Malaria Vaccine Candidate GMZ2. *Clinical Infectious Diseases* 69(8)**,** 1377-1384. doi: 10.1093/cid/ciy1087.

Diallo, M.A., Kane, B.S., Ndiaye, M., Dieng, M., Diongue, K., Badiane, A.S., et al. (2017). Plasmodium falciparum malaria co-infection with tick-borne relapsing fever in Dakar. *Malaria Journal* 16(1)**,** 24. doi: 10.1186/s12936-017-1682-6.

El Jaoudi, R., Benziane, H., Khabbal, Y., Elomri, N., Lamsaouri, J., and Cherrah, Y. (2010). Chimioprophylaxie de longue durée à la méfloquine : étude des effets indésirables. *Therapies* 65(5)**,** 439-445. doi: <https://doi.org/10.2515/therapie/2010049>.

Enger, A., Strand, O.A., Ranheim, T., and Hellum, K.B. (2004). Exflagellation of microgametocytes in Plasmodium vivax malaria: a diagnostic conundrum. *Med Princ Pract* 13(5)**,** 298-300. doi: 10.1159/000079533.

Esamai, F., Tenge, C.N., Ayuo, P.O., Ong'or, W.O., Obala, A., and Jakait, B. (2005). A Randomized Open Label Clinical Trial to Compare the Efficacy and Safety of Intravenous Quinine Followed by Oral Malarone vs. Intravenous Quinine Followed by Oral Quinine in the Treatment of Severe Malaria. *Journal of Tropical Pediatrics* 51(1)**,** 17-24. doi: 10.1093/tropej/fmh069.

Fanello, C.I., Karema, C., Ngamije, D., Uwimana, A., Ndahindwa, V., Van Overmeir, C., et al. (2008). A randomised trial to assess the efficacy and safety of chlorproguanil/dapsone + artesunate for the treatment of uncomplicated Plasmodium falciparum malaria. *Transactions of The Royal Society of Tropical Medicine and Hygiene* 102(5)**,** 412-420. doi: 10.1016/j.trstmh.2008.01.013.

Ferrer, E.B., Hansen, K.S., Gyapong, M., Bruce, J., Narh Bana, S.A., Narh, C.T., et al. (2017). Cost-effectiveness analysis of the national implementation of integrated community case management and community-based health planning and services in Ghana for the treatment of malaria, diarrhoea and pneumonia. *Malaria Journal* 16(1)**,** 277. doi: 10.1186/s12936-017-1906-9.

Gavazzi, G., Herrmann, F., and Krause, K.-H. (2004). Aging and Infectious Diseases in the Developing World. *Clinical Infectious Diseases* 39(1)**,** 83-91. doi: 10.1086/421559.

Goel, N., Gurpreet, and Swami, H.M. (2007). How to deal with emerging and re-emerging infectious diseases globally? 1.

Haque, U., Soares Magalhães, R.J., Mitra, D., Kolivras, K.N., Schmidt, W.-P., Haque, R., et al. (2011). The role of age, ethnicity and environmental factors in modulating malaria risk in Rajasthali, Bangladesh. *Malaria Journal* 10(1)**,** 367. doi: 10.1186/1475-2875-10-367.

Hazel, E., Bryce, J., and Group, t.I.-J.i.E.W. (2016). On Bathwater, Babies, and Designing Programs for Impact: Evaluations of the Integrated Community Case Management Strategy in Burkina Faso, Ethiopia, and Malawi. *The American Journal of Tropical Medicine and Hygiene* 94(3)**,** 568-570. doi: <https://doi.org/10.4269/ajtmh.94-3intro1>.

Hussain, W.M., Syed Zahid, B., Mohammad Ibrahim, F., Talal Mohammad, K., Tariq Ahmed, M., and Samar, B. (2009). Misdiagnosis of an imported case of malaria caused by Plasmodium falciparum. *The Journal of Infection in Developing Countries* 3(02). doi: 10.3855/jidc.58.

Ibrahim, M.B., Sowemimo, A.A., Sofidiya, M.O., Badmos, K.B., Fageyinbo, M.S., Abdulkareem, F.B., et al. (2016). Sub-acute and chronic toxicity profiles of Markhamia tomentosa ethanolic leaf extract in rats. *Journal of Ethnopharmacology* 193**,** 68-75. doi: <https://doi.org/10.1016/j.jep.2016.07.036>.

Iftikhar, S.A., and Roistacher, K. (1995). Indigenous Plasmodium falciparum Malaria in Queens, NY. *Archives of Internal Medicine* 155(10)**,** 1099-1101. doi: 10.1001/archinte.1995.00430100135016.

Ishengoma, D.S., Mandara, C.I., Francis, F., Talundzic, E., Lucchi, N.W., Ngasala, B., et al. (2019). Efficacy and safety of artemether-lumefantrine for the treatment of uncomplicated malaria and prevalence of Pfk13 and Pfmdr1 polymorphisms after a decade of using artemisinin-based combination therapy in mainland Tanzania. *Malaria Journal* 18(1)**,** 88. doi: 10.1186/s12936-019-2730-1.

Jande, M., Kongola, G., and Mwangi, J.W. (2012). Drug prescribing pattern in two hospitals in Mwanza, Northwest Tanzania. *East and Central African Journal of Pharmaceutical Sciences* 15(3)**,** 63-68.

Jiwani, A., Matheson, A., Kahn, J.G., Raut, A., Verguet, S., Marseille, E., et al. (2014). Integrated disease prevention campaigns: assessing country opportunity for implementation via an index approach. *BMJ Open* 4(3)**,** e004308. doi: 10.1136/bmjopen-2013-004308.

Johnson, A.S., Delisca, G., and Booth, G.S. (2013). Warm autoimmune hemolytic anemia secondary to <em>Plasmodium ovale</em> infection: A case report and review of the literature. *Transfusion and Apheresis Science* 49(3)**,** 571-573. doi: 10.1016/j.transci.2013.09.006.

Kahabuka, C., Kvåle, G., and Hinderaker, S.G. (2013). Care-Seeking and Management of Common Childhood Illnesses in Tanzania – Results from the 2010 Demographic and Health Survey. *PLOS ONE* 8(3)**,** e58789. doi: 10.1371/journal.pone.0058789.

Kam (van der), S., Salse-Ubach, N., Roll, S., Swarthout, T., Gayton-Toyoshima, S., Jiya, N.M., et al. (2016). Effect of Short-Term Supplementation with Ready-to-Use Therapeutic Food or Micronutrients for Children after Illness for Prevention of Malnutrition: A Randomised Controlled Trial in Nigeria. *PLoS Med* 13(2)**,** e1001952. doi: 10.1371/journal.pmed.1001952.

Klingelberger, C.E. (1989). It's not a viral syndrome, it's malaria. *Ann Emerg Med* 18(2)**,** 207-210. doi: 10.1016/s0196-0644(89)80118-0.

Laínez, Y.B., Wittcoff, A., Mohamud, A.I., Amendola, P., Perry, H.B., and D'Harcourt, E. (2012). Insights from community case management data in six sub-Saharan African countries. *Am J Trop Med Hyg* 87(5 Suppl)**,** 144-150. doi: 10.4269/ajtmh.2012.12-0106.

Lee, J., and Ryu, J.-S. (2019). Current Status of Parasite Infections in Indonesia: A Literature Review. *Korean J Parasitol* 57(4)**,** 329-339. doi: 10.3347/kjp.2019.57.4.329.

Macete, E., Aide, P., Aponte, J.J., Sanz, S., Mandomando, I., Espasa, M., et al. (2006). Intermittent Preventive Treatment for Malaria Control Administered at the Time of Routine Vaccinations in Mozambican Infants: A Randomized, Placebo-Controlled Trial. *The Journal of Infectious Diseases* 194(3)**,** 276-285. doi: 10.1086/505431.

Maguire, J.D., Fenton, M.E., Susanti, A.I., and Walker, J.B. (2007). Plasmodium vivax-associated acute respiratory distress syndrome after extended travel in Afghanistan. *Travel Medicine and Infectious Disease* 5(5)**,** 301-305. doi: <https://doi.org/10.1016/j.tmaid.2007.04.001>.

Mahgoub, H.M., and Adam, I. (2012). Morbidity and mortality of severe malnutrition among Sudanese children in New Halfa Hospital, Eastern Sudan. *Transactions of The Royal Society of Tropical Medicine and Hygiene* 106(1)**,** 66-68. doi: 10.1016/j.trstmh.2011.09.003.

Malchrzak, W., Rymer, W., and Inglot, M. (2018). Imported malaria caused by Plasmodium falciparum – case report. *Przeglad epidemiologiczny* 72(3)**,** 363-370. doi: 10.32394/pe.72.3.12.

Miller, J.H., Byers, M., Whiteoak, R., and Warrell, D.A. (1994). Imported Falciparum Malaria in British Troops Returning from Kenya. *Journal of the Royal Army Medical Corps* 140(3)**,** 119. doi: 10.1136/jramc-140-03-03.

Nabongo, P., Verver, S., Nangobi, E., Mutunzi, R., Wajja, A., Mayanja-Kizza, H., et al. (2014). Two year mortality and associated factors in a cohort of children from rural Uganda. *BMC Public Health* 14**,** 314. doi: 10.1186/1471-2458-14-314.

Nasveld, P., and Kitchener, S. (2005). Treatment of acute vivax malaria with tafenoquine. *Transactions of The Royal Society of Tropical Medicine and Hygiene* 99(1)**,** 2-5. doi: 10.1016/j.trstmh.2004.01.013.

Oberstaller, J., Pumpalova, Y., Schieler, A., Llinás, M., and Kissinger, J.C. (2014). The Cryptosporidium parvum ApiAP2 gene family: insights into the evolution of apicomplexan AP2 regulatory systems. *Nucleic Acids Research* 42(13)**,** 8271-8284. doi: 10.1093/nar/gku500.

Oliveira, G.A., Wetzel, K., Calvo-Calle, J.M., Nussenzweig, R., Schmidt, A., Birkett, A., et al. (2005). Safety and Enhanced Immunogenicity of a Hepatitis B Core Particle &lt;em&gt;Plasmodium falciparum&lt;/em&gt; Malaria Vaccine Formulated in Adjuvant Montanide ISA 720 in a Phase I Trial. *Infection and Immunity* 73(6)**,** 3587. doi: 10.1128/IAI.73.6.3587-3597.2005.

Pattanayak, S., and Pfaff, A. (2009). Behavior, Environment, and Health in Developing Countries: Evaluation and Valuation. *Annual Review of Resource Economics* 1. doi: 10.1146/annurev.resource.050708.144053.

Pilsczek, F.H. (2015). RESPIRATORY INFECTIONS RESEARCH IN AFGHANISTAN: BIBLIOMETRIC ANALYSIS WITH THE DATABASE PUBMED. *J Ayub Med Coll Abbottabad* 27(2)**,** 464-466.

Raj, M., Balachandran, C., Duraipandiyan, V., Agastian, P., and Ignacimuthu, S. (2012). Antimicrobial activity of Ulopterol isolated from Toddalia asiatica (L.) Lam.: A traditional medicinal plant. *Journal of Ethnopharmacology* 140(1)**,** 161-165. doi: <https://doi.org/10.1016/j.jep.2012.01.005>.

Ramesh, A., Nakielny, S., Hsu, J., Kyohere, M., Byaruhanga, O., de Bourcy, C., et al. (2019). Metagenomic next-generation sequencing of samples from pediatric febrile illness in Tororo, Uganda. *PLOS ONE* 14(6)**,** e0218318. doi: 10.1371/journal.pone.0218318.

Salako, L.A., Adio, R.A., Sowunmi, A., and Walker, O. (1990). Parenteral sulphadoxine-pyrimethamine (Fansidar): an effective and safe but under-used method of anti-malarial treatment. *Trans R Soc Trop Med Hyg* 84(5)**,** 641-643. doi: 10.1016/0035-9203(90)90131-w.

Savini, H., Gautret, P., Gaudart, J., Field, V., Castelli, F., López-Vélez, R., et al. (2013). Travel-associated Diseases, Indian Ocean Islands, 1997–2010. *Emerging Infectious Disease journal* 19(8)**,** 1297. doi: 10.3201/eid1908.121739.

Sesay, T., Denisiuk, O., Shringarpure, K.K., Wurie, B.S., George, P., Sesay, M.I., et al. (2017). Paediatric care in relation to the 2014-2015 Ebola outbreak and general reporting of deaths in Sierra Leone. *Public health action* 7(Suppl 1)**,** S34-S39. doi: 10.5588/pha.16.0088.

Tavul, L., Hetzel, M.W., Teliki, A., Walsh, D., Kiniboro, B., Rare, L., et al. (2018). Efficacy of artemether–lumefantrine and dihydroartemisinin–piperaquine for the treatment of uncomplicated malaria in Papua New Guinea. *Malaria Journal* 17(1)**,** 350. doi: 10.1186/s12936-018-2494-z.

Tedbabe, D., Marsh, D., Gobezayehu, A., Tefera, W., Osborn, G., and Waltensperger, K. (2010). Community case management improves use of treatment for childhood diarrhea, malaria and pneumonia in a remote district of Ethiopia. *Ethiopian Journal of Health Development* 23. doi: 10.4314/ejhd.v23i2.53227.

Thompson, A.A., Matamale, L., and Kharidza, S.D. (2012). Impact of climate change on children's health in Limpopo Province, South Africa. *International journal of environmental research and public health* 9(3)**,** 831-854. doi: 10.3390/ijerph9030831.

Tjitra, E., Oemijati, S., Pribadi, W., Arbani, P.R., Harianto, P.N., Popy, K., et al. (1995). Randomized comparative study of chloroquine and halofantrine in vivax malaria patients. *Medical Journal of Indonesia* 4(1). doi: 10.13181/mji.v4i1.887.

Uchiyama, H., Okamoto, A., Sato, K., Yamada, T., Murakami, S., Yoneda, S., et al. (2004). Quinine-resistant Severe Falciparum Malaria Effectively Treated with Atovaquone and Proguanil Hydrochloride Combination Therapy. *Internal Medicine* 43(7)**,** 624-627. doi: 10.2169/internalmedicine.43.624.

Ursing, J., Rombo, L., Rodrigues, A., and Kofoed, P.-E. (2016). Artemether-Lumefantrine versus Dihydroartemisinin-Piperaquine for Treatment of Uncomplicated Plasmodium falciparum Malaria in Children Aged Less than 15 Years in Guinea-Bissau – An Open-Label Non-Inferiority Randomised Clinical Trial. *PLOS ONE* 11(9)**,** e0161495. doi: 10.1371/journal.pone.0161495.

Uzma, K., Nosheen, F., Hussain, S., Tarar, M.A., Sadiq, S., and Ahmad, M. (2013). Assessment of Protein Energy Malnutrition among Children in Urban Community of Faisalabad, Pakistan. *Pakistan Journal of Nutrition* 12**,** 334-339. doi: 10.3923/pjn.2013.334.339.

Valecha, N., Looareesuwan, S., Martensson, A., Mohammed Abdulla, S., Krudsood, S., Tangpukdee, N., et al. (2010). Arterolane, a New Synthetic Trioxolane for Treatment of Uncomplicated Plasmodium falciparum Malaria: A Phase II, Multicenter, Randomized, Dose-Finding Clinical Trial. *Clinical Infectious Diseases* 51(6)**,** 684-691. doi: 10.1086/655831.

Vilkman, K., Pakkanen, S.H., Lääveri, T., Siikamäki, H., and Kantele, A. (2016). Travelers' health problems and behavior: prospective study with post-travel follow-up. *BMC Infect Dis* 16**,** 328. doi: 10.1186/s12879-016-1682-0.

Vinod, K., Talari, K., Gopalakrishnan, M., Nisar, K., and Dutta, T. (2012). Unusual presentations of vivax malaria: a report of two cases. *Journal of vector borne diseases* 49(1)**,** 49.

Waldman, A.J., and Balskus, E.P. (2018). The Human Microbiota, Infectious Disease, and Global Health: Challenges and Opportunities. *ACS Infectious Diseases* 4(1)**,** 14-26. doi: 10.1021/acsinfecdis.7b00232.

Watt, G., Loesuttiviboon, L., Jongsakul, K., Shanks, G.D., Ohrt, C.K., Karnasuta, C., et al. (1994). Efficacy and Tolerance of Extended-Dose Halofantrine for Drug-Resistant Falciparum Malaria in Thailand. *The American Journal of Tropical Medicine and Hygiene* 50(2)**,** 187-192. doi: <https://doi.org/10.4269/ajtmh.1994.50.187>.

Xu, P., Widmer, G., Wang, Y., Ozaki, L.S., Alves, J.M., Serrano, M.G., et al. (2004). The genome of Cryptosporidium hominis. *Nature* 431(7012)**,** 1107-1112. doi: 10.1038/nature02977.

Yakoob, M.Y., Theodoratou, E., Jabeen, A., Imdad, A., Eisele, T.P., Ferguson, J., et al. (2011). Preventive zinc supplementation in developing countries: impact on mortality and morbidity due to diarrhea, pneumonia and malaria. *BMC Public Health* 11(3)**,** S23. doi: 10.1186/1471-2458-11-S3-S23.

Zoungrana, A., Coulibaly, B., Sié, A., Walter-Sack, I., Mockenhaupt, F.P., Kouyaté, B., et al. (2008). Safety and Efficacy of Methylene Blue Combined with Artesunate or Amodiaquine for Uncomplicated Falciparum Malaria: A Randomized Controlled Trial from Burkina Faso. *PLOS ONE* 3(2)**,** e1630. doi: 10.1371/journal.pone.0001630.
